# Restructuring the Cellular Responses: Connecting Microbial Intervention With Ecological Fitness and Adaptiveness to the Maize (*Zea mays* L.) Grown in Saline–Sodic Soil

**DOI:** 10.3389/fmicb.2020.568325

**Published:** 2021-02-12

**Authors:** Shailendra Singh, Udai B. Singh, Mala Trivdi, Deepti Malviya, Pramod K. Sahu, Manish Roy, Pawan K. Sharma, Harsh V. Singh, M. C. Manna, Anil K. Saxena

**Affiliations:** ^1^Plant-Microbe Interaction and Rhizosphere Biology Lab, ICAR-National Bureau of Agriculturally Important Microorganisms, Kushmaur, India; ^2^Amity Institute of Biotechnology, Amity University Uttar Pradesh, Lucknow, India; ^3^Soil Biology Division, ICAR-Indian Institute of Soil Science, Bhopal, India

**Keywords:** seed biopriming, rhizosphere microorganisms, Maize (*Zea mays* L), saline–sodic soil, antioxidant enzymes, salt tolerance, High-Affinity K+ Transporter, *Sodium/hydrogen exchanger*

## Abstract

Salt stress hampers plant growth and development. It is now becoming one of the most important threats to agricultural productivity. Rhizosphere microorganisms play key roles in modulating cellular responses and enable plant tolerant to salt stress, but the detailed mechanisms of how this occurs need in-depth investigation. The present study elucidated that the microbe-mediated restructuring of the cellular responses leads to ecological fitness and adaptiveness to the maize (*Zea mays* L.) grown in saline–sodic soil. In the present study, effects of seed biopriming with *B. safensis* MF-01, *B. altitudinis* MF-15, and *B. velezensis* MF-08 singly and in consortium on different growth parameters were recorded. Soil biochemical and enzymatic analyses were performed. The activity and gene expression of High-Affinity K^+^ Transporter (*ZmHKT-1*), Sodium/Hydrogen exchanger 1 (*zmNHX1*), and antioxidant enzymes (*ZmAPX1.2*, *ZmBADH-1*, *ZmCAT*, *ZmMPK5*, *ZmMPK7*, and *ZmCPK11*) were studied. The expression of genes related to lateral root development (*ZmHO-1*, *ZmGSL-1*, and *ZmGSL-3*) and root architecture were also carried out. Seeds bioprimed with consortium of all three strains have been shown to confer increased seed germination (23.34–26.31%) and vigor indices (vigor index I: 38.71–53.68% and vigor index II: 74.11–82.43%) as compared to untreated control plant grown in saline–sodic soil at 30 days of sowing. Results indicated that plants treated with consortium of three strains induced early production of adventitious roots (tips: 4889.29, forks: 7951.57, and crossings: 2296.45) in maize compared to plants primed with single strains and untreated control (tips: 2019.25, forks: 3021.45, and crossings: 388.36), which was further confirmed by assessing the transcript level of *ZmHO-1* (7.20 folds), *ZmGSL-1* (4.50 folds), and *ZmGSL-3* (12.00 folds) genes using the qPCR approach. The uptake and translocation of Na^+^, K^+^, and Ca^2+^ significantly varied in the plants treated with bioagents alone or in consortium. qRT-PCR analysis also revealed that the *ZmHKT-1* and *zmNHX1* expression levels varied significantly in the maize root upon inoculation and showed a 6- to 11-fold increase in the plants bioprimed with all the three strains in combination. Further, the activity and gene expression levels of antioxidant enzymes were significantly higher in the leaves of maize subjected seed biopriming with bioagents individually or in combination (3.50- to 12.00-fold). Our research indicated that *ZmHKT-1* and *zmNHX1* expression could effectively enhance salt tolerance by maintaining an optimal Na^+^/K^+^ balance and increasing the antioxidant activity that keeps reactive oxygen species at a low accumulation level. Interestingly, up-regulation of *ZmHKT-1*, *NHX1*, *ZmHO-1*, *ZmGSL-1*, and *ZmGSL-3* and genes encoding antioxidants regulates the cellular responses that could effectively enhance the adaptiveness and ultimately leads to better plant growth and grain production in the maize crop grown in saline–sodic soil.

## Introduction

Soil salinity and sodicity are major abiotic stresses, causing considerable yield loss in a wide range of crops worldwide. An estimate indicated that 7% of the world’s total arable land (955 million ha) is affected by salt, and in the next 25 years, this may reach up to 30% of the total cultivable land (Flowers et al., 1997; Singh S. et al., 2019). Further, it is estimated that up to 50% of total cultivable land will be lost due to salinity and sodicity by 2050 (Shahbaz and Ashraf, 2013). In India, 7 million ha of land is affected by salt stress, while the Indo-Gangatic Plain (IGP) region alone has approximately 2.7 million ha of area being affected by soil salinity and sodicity (Singh S. et al., 2019). These soils are usually unfit for crop production due to high pH, low organic matter content, and high concentrations of soluble salts such as Na_2_CO_3_, NaHCO_3_, NaCl, etc., and together with sufficient exchangeable sodium, they cause poor soil physio-biochemical characteristics (Das, 2009). Excessive salt/Na^+^ in the soil solution affects absorption of water and mineral nutrient and, therefore, causes seedling mortality by pulling water from the root system (exo-osmosis) and disrupting cellular function (Das, 2009; Shrivastava and Kumar, 2015; Singh et al., 2016a; Singh S. et al., 2019).

Maize (*Zea mays* L.) is considered as one of the most important cereal crops after rice and wheat all over the world (Nuss and Tanumihardjo, 2010). It is grown under a wide spectrum of soil and climatic conditions. In addition, maize is moderately sensitive to salt stress and is responsible for significant loss of crop yield worldwide (Anonymous, 2018; Singh S. et al., 2019). Soil salinity and sodicity reduce seed germination, early crop growth by suppressing leaf initiation and expansion, as well as internode growth and poor crop establishment. Further, salt stress leads to membrane damage, reduction in leaf relative water content, denaturation of proteins, accumulation of oxidizing substances, inactivation of enzymes, and decline of photosynthesis rate, which ultimately leads to stunted growth and low yield in maize (Apse et al., 1999; Shrivastava and Kumar, 2015; Singh S. et al., 2019). Therefore, salinity and sodicity proved to be two of the most serious threats to maize production (Singh S. et al., 2019).

To withstand salt stress, plants have evolved sophisticated and complex signaling mechanisms/response pathways resulting in adaptive responses through morphological and physio-biochemical changes (Gholami et al., 2009; Jiang et al., 2016, 2017; Komis et al., 2018). Further, roots are the first and foremost part of the plants exposed to any stress. The development of root architecture is in general regulated by genetic make-up of the plants including inbred lines or cultivars and environmental factors including abiotic and biotic stresses (Zimmermann et al., 2010; Zhang et al., 2018). Several studies reported that a number of genes have been found to be involved in lateral root development in maize (Zimmermann et al., 2010; Ding et al., 2013; Zhang et al., 2018). In general, several research findings reported the role of microbial inoculants on the root growth and biomass accumulation (Singh et al., 2016a, b, 2019). However, none of the studies showed the involvement of rhizospheric microbes on the activation of genes involved in lateral root development in maize under salt-stressed conditions.

To exploit the salt-stressed soil, new and more efficient ways to increase crop yield are important for sustainable crop production and food security, particularly in saline–sodic soil of India (Das, 2009; Shrivastava and Kumar, 2015). Plant breeders have tried to develop salt-tolerant cultivars in many crops including maize. However, none of the maize cultivars are commercially available so far, which possess high degree of salt tolerance (Farooq et al., 2015). Therefore, we still need such donor parents having quantitative trait loci (QTLs) or gene(s) with salt tolerance to prevent losses to our maize production (Singh S. et al., 2019). Several alternatives have been utilized to improve salt tolerance, and among them, exploitation of salt-tolerant compatible microbial inoculants is an emerging approach to ameliorate the toxic effects of salt in crop plants (Ashraf and Foolad, 2007; Agbodjato et al., 2016; Latef and Tran, 2016). In the recent past, attention has been given to identify and utilize the consortia of compatible salt-tolerant rhizospheric microorganisms (STRM) that can mediate induced systemic tolerance (IST) to sustain and improve plant growth under such stressful conditions (Gaiero et al., 2013; Egamberdieva et al., 2019; Irfan et al., 2019). The salt tolerance responses are triggered by STRM or STRM-derived elicitors/signaling molecules and eventually lead to extensive transcriptional reprogramming (Munns and Tester, 2008; Pitann et al., 2013; Kifle and Laing, 2016a, b). Numerous salt tolerance-related genes are up-regulated in the plant under salt stress and primed with STRM (Sandhya et al., 2010; Shi et al., 2010; Wang et al., 2010; Szoboszlay et al., 2015; Sun et al., 2015; Soares et al., 2018). Considerable efforts have been made to utilize plant growth-promoting (PGP) rhizosphere microorganisms for enhanced salt tolerance in many crops (Sandhya et al., 2010; Singh et al., 2016a; Tiwari et al., 2018; Singh S. et al., 2019; Vaishnav et al., 2020). More importantly, these studies have shown the rhizosphere microbe-mediated reprogramming in the cellular physiological networks, genetic base of salt tolerance, regulation of antioxidant defense systems, and photosynthesis, and finally alleviate the detrimental effects of salt in many plant species in a fragmented way (Yamaguchi and Blumwald, 2005; Bais et al., 2006; Liu et al., 2006; Young et al., 2013; Mahmood et al., 2016; Bokhari et al., 2019; Wang et al., 2020). Though the rhizosphere microbiome constitutes a rich gene pool, selecting rhizospheric partners from the same plant species would offer a competitive advantage for the microbe to succeed (Mahoney et al., 2017; Singh S. et al., 2019; Molina-Montenegro et al., 2020). Moreover, information regarding the effects of native rhizobacteria on physiological and antioxidant machinery of salt-stressed maize plants is limited and needs to be further explored. The objective of the present study was to elucidate the microbe-mediated physio-biochemical and molecular mechanisms that provide salt tolerance, ecological fitness, and adaptiveness to maize plants grown in saline–sodic soil. This systematic investigation provided novel insights to better understand the mechanisms of the microbe-induced plant to salt tolerance and enhancement in the maize production under saline–sodic soil.

## Materials and Methods

### Isolation and Characterization of Bacterial Isolates

Twenty-five samples of maize rhizosphere soil were collected from different parts of Uttar Pradesh and Madhya Pradesh (India) during 2015–2016 and brought to the laboratory (Supplementary Table 1). Soil samples were sieved (2 mm pore size), air dried under shade to remove the excess moisture, and used for isolation. Two hundred fifty bacterial isolates were isolated from rhizosphere soil by plating serial decimal dilution on HiChrome Bacillus Agar (HiMedia, India), and all the isolates were propagated and kept on Nutrient Agar (NA) medium at 4°C until further use.

These isolates were evaluated for salt tolerance capability on NA supplemented with different concentrations of NaCl (1–10%) as per methods described by Singh et al. (2015). Further, the isolates tolerating above 5% of NaCl were screened for P (Nautiyal, 1999), K (Rajawat et al., 2016), and Zn (Sharma et al., 2014) solubilization using the standard protocols. Based on salt tolerance, and P, K, and Zn solubilization, 154 potential isolates were selected for identification at the molecular level. The selected isolates were further screened for their PGP traits, *viz.*, indole acetic acid (Brick et al., 1991), siderophore (Schwyn and Neilands, 1987), and ammonia (Dey et al., 2004) production. The production of HCN, H_2_O_2_, urease, catalase, and starch hydrolysis test was performed as per the methods described in Whitman et al. (2012). However, protease estimation was carried out as per the methods described by Boller and Mauch (1988).

### 16S rRNA Gene Sequencing and Phylogenetic Analysis

Molecular identification of the shortlisted isolates was conducted by DNA extraction and 16S rRNA gene amplification using universal primers pair 27F and 1492R 42 (Edwards et al., 1989). The 16S rRNA gene amplicons were further subjected to restriction endonuclease digestion by using endonucleases *Hae-III*, *Alu-1*, and *Msp-1*. The digested amplicons were separated in 2.5% agarose gel added with ethidium bromide, and electrophoresis was done at 45 V for 1.5 to 2 h in 1 × TAE buffer. Grouping was done using NTSYSpc, version 2.02 software and at least one representative isolate was selected for sequencing of 16S rRNA gene amplicon. RFLP pattern revealed that all 154 isolates fall into 23 different clusters. From each group, best-performing isolates were selected and ended with a total of 50 isolates. In this way, 16S rRNA gene of 50 isolates was sequenced through Eurofin Pvt. Ltd. (India) and the sequence similarity was matched using the EzBiocloud database for identification. Phylogenetic analysis was carried out using the Molecular Evolutionary Genetics Analysis (MEGA-X) tools and 16S rRNA gene sequences were submitted to the NCBI GenBank.

### *In planta* Assay

#### Experimental Setup

The experiments consisted of five different treatments: T_1_—*B. safensis* MF-01, T_2_—*B. altitudinis* MF-15, T_3_—*B. velezensis* MF-08, T_4_—*B. safensis* MF-01 + *B. velezensis* MF-08 + *B. altitudinis* MF-15 (1:1:1), and T_5_—control (untreated). Each treatment consisted of 10 replications under nethouse conditions, whereas 5 replications were maintained under field conditions in a randomized block design (RBD). The nethouse experiments and field experiment were conducted during *Summer* (March to May) and *Kharif* season (June to August) in 2017–2018.

#### Soil Collection, Preparation, and Analysis

To lay out the pot experiments, soil was collected from Research Farm, ICAR-National Bureau of Agriculturally Important Microorganisms (ICAR-NBAIM), Kushmaur, air dried in shade, sieved (2 mm), and brought to the laboratory. The soil belongs to texture class Dystric Eutrudepts (Inceptisols). Further, soil was mixed with nitrogen, phosphorus, and potassium at 150, 80, and 40 kg ha^–1^, respectively. Soil was filled in HiDispo polythene bags (HiMedia, India) and autoclaved twice for 30 min (121°C at 15 PSI) at 24-h intervals. The autoclaved soil was kept as such for 3–5 days under shade to maintain the natural ionic equilibrium inside the soil. The initial soil properties were analyzed using standard protocols and presented in Supplementary Table 2.

### Development of Liquid-Based Bioformulation

The new medium (liquid base) was designed and developed by US and his team and was taken for preparation of bioformulations (composition was not shown due to IP protection). Broadly, liquid-based formulation of *Bacillus* spp. was developed using nutrient broth constituent as a base (peptone, 5 *g*; beef extract, 3 *g*; NaCl, 5 *g*; water, 1000 ml; and pH at 25°C: 7.3 ± 0.2). After 96 h of growth, 15% sterile glycerol along with 0.01% PVP was added to the formulation. The growth kinetics of selected strains was tested and found to be almost the same. For preparation of bioformulations, all the three strains were grown separately in the liquid medium till CFU count reached 2.5 × 10^8^ and were taken as primary inoculums/mother culture. Further, 1 ml of primary inoculums was added in the 100-ml liquid medium for preparation of working formulation. During consortium development, primary inoculums were mixed in a 1:1:1 ratio to develop bioformulation. The selected strains were inoculated singly and in combination of the three in the medium, and incubated for 5 days in the incubator shaker at 150 RPM at 28°C. After 5 days, 1 ml of culture suspension was taken and colony-forming units were measured by plating serial decimal dilution on NA medium. The CFU count of the final product was 2.24 × 10^8^ at the time of application.

### Planting Material and Growth Conditions

Maize seeds (*cv*. Sachin 777) used in this study were procured from an open market. Surface sterilization was done with sodium hypochlorite (NaOCl, 1%) for 2 min, followed by three washing cycles with sterile distilled water under aseptic conditions. Maize seeds were bioprimed with liquid bioformulation (10 ml kg^–1^ seed suspended in 40 ml of water containing 0.01% gum acasia and 0.01% trehalose) and incubated overnight under the shade (Singh et al., 2020). Seeds treated with sterile liquid bioformulation containing 15% sterile glycerol along with 0.01% PVP served as control. The bioprimed and untreated control seeds were sowed in the plastic post (20 × 20 cm) containing sterilized experimental soil (5 kg). Two plants were maintained in each pot and a control set (untreated) was maintained in each experiment. The pots were watered manually on alternate days to maintain the moisture content (60%).

Field experiments were conducted at Research Farm, ICAR-NBAIM, Kushmaur in *Summer* and *Kharif* maize. The experiments consisted of five different treatments in five replicates. The size of the individual plot was 5 × 4 m^2^ with a border space of 1 m. The bioprimed seeds were sown in the field manually in the evening hours with a spacing of 45 × 30 cm (row-to-row and plant-to-plant spacing). The average mean temperature and relative humidity during the experimentation in the *Summer* maize were 27.36°C and 56–61%, respectively, whereas it was 26.25°C and 76–81%, respectively, in the *Kharif* maize.

### Effect of Seed Biopriming on Seed Germination and Vigor Indices

Effects of biopriming on seed germination (%) and vigor indices were studied in sterile sand and soil mixture (1:3) under nethouse conditions as per methods described by Singh et al. (2016b). In brief, for seed germination (%) test, 300 seeds were sterilized with sodium hypochlorite (1%) and washed thrice in sterile distilled water. Thereafter, sterilized seeds were bioprimed with selected strains *B. safensis* MF-01, *B. altitudinis* MF-15, and *B. velezensis* MF-08 individually and in combination of three as per treatments and sown in tray (45 × 30 × 10 cm) containing sterile sand and soil mixture. After 15 days of sowing, percent germination was calculated. To measure the vigor indices, sterile seeds (3) were planted in each pot (20 cm diameter) containing sterile sand and soil mixture (3 kg). After sowing, the pots were irrigated on alternate days with sterile water throughout the experimental period. After 30 days of sowing, the vigor indices were calculated as per methods described by Singh et al. (2016b). The growth conditions in the nethouse were as follows: 14/10 h day/night photoperiod, 38/29°C day/night temperature, and 50/60% day/night humidity in *Summer* maize. Growth conditions were 13/11 h day/night photoperiod, 34/25°C day/night temperature, and 70/85% day/night humidity in *Kharif* maize.

### Effect of Seed Biopriming on Root Architecture and Root Development

To analyze the effect of seed biopriming on root architecture, bioprimed seed (2 No.) was planted in the pots containing sterile sand soil mixture as discussed in the section *Effect of seed biopriming on seed germination and vigor indices*. After seed germination, a single seedling was maintained in each pot throughout the experiment. Thereafter, plants were allowed to grow for the next 30 days. On the 30th day, plants were uprooted gently and brought to the laboratory. The roots were washed carefully under running tap water and clean roots were scanned (Regent Instrument, Canada). The scanned images were analyzed using image analysis software “WinRhizo Pro 2017” (Client# IN1803202) to study the different parameters of root architecture. To see the root colonization potential of selected strain(s), plants were uprooted gently and washed in running tap water, the root samples were fixed with the help of glutaraldehyde (2.5%) and formaldehyde (37%; 1:1), and microscopy was done using a scanning electron microscope (Hitachi S-3400N, United States) as per methods described by Singh U. B. et al. (2019).

A quantitative real time-PCR (qPCR) analysis was performed to investigate the expression of 11 genes conferring secondary root development, plant adaptiveness, and salinity tolerance in maize. For qPCR analyses, root samples were collected after 30 days of sowing. The root samples were quick-frozen in liquid nitrogen and ground, and total RNA was isolated using RNA isolation kit (Agilent, India) following the manufacturer’s protocol. One microgram of RNA was used to synthesize cDNA with oligo-dT using cDNA Synthesis Kit (BioRAD, India) according to the manufacturer’s instructions/protocols. The concentration of cDNA was determined using Nanodrop 2000c (Thermo Scientific, United States). The housekeeping gene actin was used as an endogenous standard to normalize the quantitative expression data of *ZmHO-1* (*Zea mays Haem Oxygenase-1*), *ZmGSL-1* (*Zea mays Gibberellic Acid Stimulated-Like 1*), and *ZmGSL-3* (*Zea mays Gibberellic Acid Stimulated-Like 3*). The gene expression was analyzed using gene-specific primers (Table 1). The qRT-PCR was performed using the SYBR Green master mix (Thermo Scientific) on the BioRAD Real-Time PCR System (MJ MiniOpticon System, BioRAD). The specificity of the amplification was verified by melting-curve analysis. Three replications were performed for each sample. The relative transcription levels were calculated using the 2^–ΔΔC^_T_ method (Livak and Schmittgen, 2001).

**TABLE 1:** Primers used in qRT-PCR analyses.

S. No.	Gene name	Primer sequences	References
1.	**Housekeeping gene**		
	*ZmActin*-RT-F	CTGAGGTTCTATTCCAGCCATCC	Jiang et al., 2018
	*ZmActin*-RT-R	CCACCACTGAGGACAACATTACC	Jiang et al., 2018
2.	**High-Affinity K^+^ Transporter**		
	*ZmHKT1*-RT-F	TCGGCTCTGGACCTACTCTT	Zhang et al., 2017
	*ZmHKT1*-RT-R	ACGACGACGACTCTGCTCTA	Zhang et al., 2017
3.	**Sodium/hydrogen exchanger 1**		
	*ZmNHX1*-RT-F	ATGCAGGGTTCCAAGTGAAG	Jiang et al., 2016
	*ZmNHX1*-RT-R	AATATTGCCCCAAGTGCAAG	Jiang et al., 2016
4.	**Genes related to lateral root development**		
	*ZmHO-1*-RT-F	ACACTGTTGGCTGATCCAGT	Zhang et al., 2018
	*ZmHO-1*-RT-R	AAACGTATCTGGGGGAGGGA	Zhang et al., 2018
	*ZmGSL-1*-RT-F	CTAATTTGCTGCGCGGCAATG	Zhang et al., 2018
	*ZmGSL-1*-RT-R	CACTTGCGGCAGAAGAAGAG	Zhang et al., 2018
	*ZmGSL-3*-RT-F	GCTCTGCGGCAGCAGGTGAAG	Zimmermann et al., 2010
	*ZmGSL-3*-RT-R	GATGTTCCTCATCAATCCGGGG	Zimmermann et al., 2010
5.	**Antioxidant enzymes**		
	*ZmAPX-1.2*-RT-F	GGCAAGCAGATGGGTTTGA	Phillips et al., 2018
	*ZmAPX-1.2*-RT-R	CTCCACAAGAGGGCGGAAGA	Phillips et al., 2018
	*ZmBADH-1*-RT-F	ATTGGGGTTGTTGGACTGATCACTC	Phillips et al., 2018
	*ZmBADH-1*-RT-R	TGGGAATGTGAGGATAATGGAGCAC	Phillips et al., 2018
	*ZmCAT*-RT-F	TCAAGCCGAATCCAAAGACCA	Phillips et al., 2018
	*ZmCAT*-RT-R	TCGAGCAAGCATTTCACACCA	Phillips et al., 2018
	*ZmMPK-5*-RT-F	GATTATCAGTAGCCAAAGTTCAA	Jiang et al., 2016
	*ZmMPK-5*-RT-R	ACACCGTCACCAGCTTTTAATC	Jiang et al., 2016
	*ZmMPK-7*-RT-F	CCAGTAGCCAAAGTTCGGTTC	Jiang et al., 2016
	*ZmMPK-7*-RT-R	TACAGACAACACCGAGAAGTACTTA	Jiang et al., 2016
	*ZmCPK-11*-RT-F	AGAACGAAATCCAGGCTCTAATG	Jiang et al., 2016
	*ZmCPK-11*-RT-R	ATTCGCGACATGCTTGTGAC	Jiang et al., 2016

### Effect of Seed Biopriming and Microbial Intervention on Physio-Biochemical Parameters and Antioxidant Enzymes

The quantitative estimation was done to see the effects of seed biopriming on physio-biochemical properties and antioxidant enzymes in the leaves of plants bioprimed with selected strain(s) at 30 days of sowing. Total chlorophyll, carotenoids, total soluble sugar, and total protein were estimated quantitatively according to Sadasivam and Manickam (1996). Plants tend to overproduce proline, phenolics, flavonoids, and antioxidant enzymes under stress conditions. The synthesis and accumulation of total proline, total phenolics, and total flavonoids in the maize leaves were assayed as per methods described by Thimmaiah (2012). The activity of catalase and peroxidase was assayed according to Sadasivam and Manickam (1996). However, the activity of superoxide dismutase (SOD) was assayed spectrophotometrically as per methods described by Singh S. et al. (2019).

To investigate whether seed biopriming with selected strain(s) up-/down-regulate the antioxidant gene expression such as *ZmAPX1.2* (*Ascorbate peroxidase 1.2*), *ZmBADH*-1 (*betaine aldehyde dehydrogenase-1*), *ZmCAT* (*Catalase*), *ZmMPK5*, *ZmMPK7*, *ZmCPK11*, *zmHNX1* (*Zea mays Sodium/hydrogen exchanger 1*), and *zmHKT1* (*Zea mays high-affinity K^+^ transporter 1*) was studied in the maize plants under salt-stressed conditions. Total mRNA was extracted from maize leaves at 15 days of sowing; cDNA synthesis and quantification of cDNA were done as per methods described in the section *Effect of seed biopriming on root architecture and root development*. The qRT-PCR was performed using the SYBR Green master mix (Thermo Scientific) and primer pairs (Table 1) on the BioRAD Real-Time PCR System (MJ MiniOpticon system, BioRAD). The specificity of the amplification was verified by melting-curve analysis. The relative transcription levels were calculated using the 2^–ΔΔC^_T_ method (Livak and Schmittgen, 2001) and housekeeping gene actin was used as an endogenous standard to normalize the quantitative expression data. Three replications were performed for each sample.

### Effect of Seed Biopriming and Microbial Intervention on Plant Growth Attributes, Physiological Traits, and Yield

To determine the effects of seed biopriming on plant growth attributes, physiological traits, and yield, and pot and field experiments were conducted on maize grown in saline–sodic soil. From nethouse experiments, five plants were selected randomly and uprooted from each treatment to observe average shoot and root length, average number of leaves per plant, and average fresh and dry biomass of shoot and root in the *Summer* and *Kharif* maize at 45 DAS. However, five plants were selected from the field experiments to measure the average plant height, average number of leaves, and dry biomass of shoot and root in the in the *Summer* and *Kharif* maize at 45 DAS. To see the effects of seed biopriming and microbial intervention on physiological traits, five plants were sampled randomly and we measured the leaf area index, mean crop growth rate, mean relative growth rate, and mean assimilation rate in the *Summer* and *Kharif* maize grown under field conditions at 45 DAS as per methods described by Rajput et al. (2017). Further, the effects of microbial intervention on yield-attributing traits such as average number of cobs/plant, average number of grain/cob, test weight (weight of 1000 grains), and yield/plot were measured in the *Summer* and *Kharif* maize grown under field conditions at harvest.

### Effect of Seed Biopriming and Microbial Intervention on Soil Enzymes, Microbial Biomass Carbon, and Soil Respiration

Rhizosphere soil samples were collected from *Kharif* maize grown under nethouse experiments to measure the activity of soil enzymes such as dehydrogenase, alkaline phosphatase, acid phosphatase, protease, cellulase, invertase, microbial biomass carbon (MBC), and soil respiration at 45 DAS. The quantitative estimation of dehydrogenase activity (μg TPF/g of dry soil/24 h) in the rhizospheric soil samples was carried out using spectrophotometric method as given by Casida et al. (1964). The alkaline phosphatase activity (μg PNP/g of dry soil/2 h) was assayed as per methods described by Tabatabai and Bremner (1969). Briefly, soil samples (1 *g*) were taken in a 50-ml Erlenmeyer flask containing 0.2 ml of toluene, 4 ml of modified universal buffer, and 1 ml of *p*-nitrophenol phosphate solution and incubated at 37°C for 1 h. Further, calcium chloride (1 ml) and sodium hydroxide (4 ml) were added in the flasks. The intensity of yellow color (*p*-nitrophenol) developed in the filtrate was measured colorimetrically at 440 nm. Further, to estimate acid phosphatase activities in the soils, samples were incubated with Na *p*-nitrophenyl phosphate at 37°C in universal buffer (pH 6.5) for 1 h by adding 0.25 ml of toluene to control microbial growth (Tabatabai and Bremner, 1969). The protease activity in the soil was determined by following the protocols described by Rejšek et al. (2008). Briefly, 0.05 M tris–HCl buffer (2 ml) and 1% casein (2 ml) were added in the soil samples (1 *g*) and incubated at 49°C for 2 h. Trichloroacetic acid (1 ml) was added to stop the reaction. Further, 1 ml of supernatant was added to the Na_2_CO_3_ and CuSO_4_ solution and mixed properly. Thereafter, Folin–Ciocalteau reagent (1 ml) was added and the concentration of aromatic amino acids was determined spectrophotometrically at 648 nm. L-Tyrosine was taken as standard and activity was denoted as μg tyrosine g^–1^ of dry soil h^–1^.

To estimate the cellulose activity in the soil (μg glucose g^–1^ of dry soil h^–1^), the soil samples (5 *g*) were taken into an Erlenmeyer flask (50 ml) containing 0.5 ml of toluene, 10 ml of acetate buffer, and 10 ml carboxymethyl cellulose solution (Pancholy and Rice, 1973). The flasks were incubated at 30°C for 24 h. The reducing sugar content in the samples was determined by Nelson–Somogyi’s method by using D-glucose as standard. The invertase activity (mg glucose g^–1^ of dry soil h^–1^) in the maize rhizosphere soil was determined as per methods described by Gianfreda et al. (1995) with slight modifications. Briefly, the soil samples (1 *g*) were taken into an Erlenmeyer flask (50 ml) containing saccharose (5 ml) and sodium acetate buffer, incubated at 30°C for 1 h, and centrifuged at 3,500 *g* for 10 min. The concentration of sugar produced from saccharose was determined by Nelson–Somogyi’s reagent. D-glucose was taken as standard.

To estimate the MBC, soil samples (10 *g*) were added into the beakers containing 25 ml of chloroform and fumigated in airtight desiccators for 5 min. Thereafter, 0.5 M K_2_SO_4_ (40 ml) was added to the beaker and shaken for 30 min. Further, 8 ml of the filtrate was taken into conical flask (500 ml) containing 2 ml of K_2_Cr_2_O_7_ (0.2 N), 10 ml of concentrated sulfuric acid, and 5 ml of orthophosphoric acid, and the content was refluxed at 100°C for 30 min. After cooling the sample, ferroin indicator was added and titrated against standard ferrous ammonium sulfate to obtain a brick-red end point. The organic carbon was determined in non-fumigated soil by following a similar method for determining MBC in terms of mg kg^–1^ of dry soil. However, soil respiration was measured as per methods described by Bekku et al. (1995). Briefly, for each replication, 50 *g* of moist soil was incubated in a 500-ml jar (along with two blanks) with an alkali trap containing 20 ml of 1 *N* NaOH to capture CO_2_. To measure soil respiration, alkali traps after 10 days of incubation were drawn out of the jars. Amount of CO_2_ trapped was determined by back titration of the 1 *N* NaOH with 0.5 *N* HCl at pH 8.3 in the presence of saturated BaCl_2_ using phenolphthalein indicator. The following equation was used to assess CO_2_ evolved:

(1) C⁢O⁢2⁢C⁢e⁢v⁢o⁢l⁢v⁢e⁢d=(A⁢B)×N× 6⁢…

where A and B are the volumes (ml) of HCl consumed for titrating 10 ml 1 *N* NaOH of flask without soil and flask with soil, respectively; *N* is the normality of HCl, and six is the equivalent weight of C. The value was expressed in mg CO_2_-C (100 *g*)^–1^ (10 day)^–1^ on dry weight basis.

### Effects of Seed Biopriming and Microbial Intervention on Na^+^, K^+^, and Ca^2+^ Uptake

Five plants were sampled to measure the uptake of Na^+^, K^+^, and Ca^2+^ from the *Kharif* maize grown under field experiments at harvest. The quantitative measurements of Na^+^, K^+^, and Ca^2+^ content in the root and shoot were performed as per methods described by Zhang et al. (2008) using the inductively coupled plasma spectrograph (Optima 2100DV, Perkin Elmer).

### Statistical Analysis

Nethouse and field experiments were carried out in complete RBD in 10 and 5 replications, respectively. Data were subjected to analysis of variance (ANOVA) and least significant difference (LSD) at *p* ≤ 0.05 using SAS 9.2 version. Data were compared with Duncan’s multiple range test at *p* ≤ 0.05. Graphs were prepared using statistical software Origin 9.0.

## Results

### Microbial Strains

**FIGURE 1:**
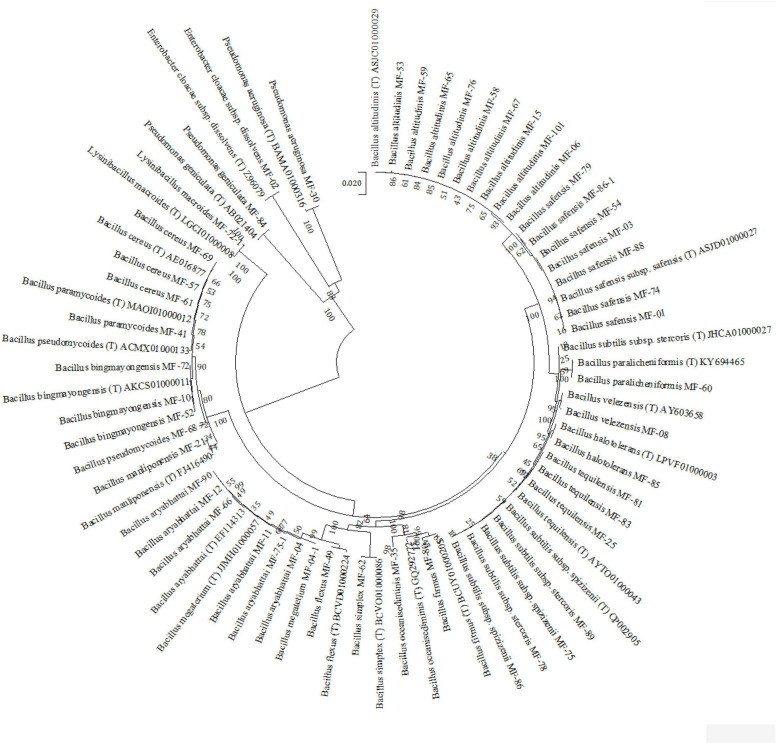
Neighbor joining tree derived by CLUSTAL W and MEGA X using analysis of 16S rRNA sequences of bacterial strains isolated from maize rhizosphere at different growth stages. The numbers at nodes indicate bootstrap support values, as calculated by MEGA X.

During the course of exploration and isolation of microbial strains for alleviation of salt stress, 250 strains were isolated from different soil samples collected. These strains were screened for salt tolerance (NaCl 1–10%). Based on the salt tolerance (above 5% NaCl), P, K, and Zn solubilization, and PGP traits, 154 potential isolates were selected for identification at the molecular level. Molecular characterization of 154 selected strains was carried out based on 16S rRNA gene amplification and its RFLP pattern with a set of three endonuclease restriction enzymes *Hae-III*, *Alu-1*, and *Msp-1*. A total of 50 strains were selected as representatives based on RFLP clustering at 70% similarity level and the PGP traits they have shown (Supplementary Table 3). These 50 strains were sequenced by Sanger’s di-deoxy nucleotide sequencing method, and identification was done based on percentage similarity (EzBiocloud, a public database of type strains) by BLAST homology (Supplementary Table 4). These sequences were submitted to NCBI GenBank and accession numbers were obtained. Based on BLAST homology results, 23 different species including 20 species of *Bacillus* and one each of *Pseudomonas aeruginosa*, *P. geniculata*, and *Enterobacter cloacae* subsp. *dissolvens* were reported. It was further observed that *Bacillus altitudinis* was the most dominant species with nine strains followed by *B. safensis* with seven strains and *B. aryabhattai* with six strains (Figure 1). Therefore, on the basis of salt tolerance (>8% NaCl), P, K, and Zn solubilization, and other PGP traits tested *in vitro*, the most promising strains *B. safensis* MF-01, *B. altitudinis* MF-15, and *B. velezensis* MF-08 were selected for further *in planta* experimentation on maize grown in saline–sodic soil.

### Effect of Microbial Intervention on Seed Germination and Seedling Vigor

Effects of seed biopriming and microbial intervention on seed germination (%), vigor index I, and vigor index II were studied under nethouse conditions after 30 DAS. All the three strains, i.e., *B. safensis* MF-01, *B. altitudinis* MF-15, and *B. velezensis* MF-08 individually or in consortium, were found to increase percent seed germination in *Summer* and *Kharif* maize. Results showed that maximum germination was recorded in the seeds treated with *B. safensis* MF-01, *B. altitudinis* MF-15, and *B. velezensis* MF-08 in combination compared to individual inoculation in both *Summer* (94.36%) and *Kharif* (96.25%) maize. However, no significant difference was recorded for the percent seed germination in case of individual inoculation. The least seed germination (%) was recorded in untreated control (Table 2). Similarly, the highest vigor index I and vigor index II were recorded in the plants inoculated with consortium of all three strains in *Summer* (4550.33 and 405.22, respectively) and *Kharif* (4675.29 and 444.50, respectively) maize. However, significant differences in the vigor index I and vigor index II were recorded in the plants treated with either bioinoculant individually in *Summer* and *Kharif* maize at 30 DAS. The lowest vigor index I and vigor index II were recorded in the untreated control plants in *Summer* (3280.43 and 222.75, respectively) and *Kharif* (3042.25 and 255.75, respectively) maize (Table 2). It was further observed that *Kharif* maize showed higher vigor indices compared to *Summer* maize. None of the microbial strains tested individually or in combination had a phytotoxic as well as an adverse effect on maize plants (visual observations, data not shown) grown in *Summer* and *Kharif* season. It was concluded that consortium of all three strains gave significantly higher percentage of seed germination and vigor indices as compared to treatments and untreated control.

**TABLE 2:** Effects of seed biopriming and microbial intervention on germination (%) and vigor indices in maize grown in *Summer* and *Kharif* season under nethouse experiments at 30 days of sowing.

Treatments	Germination (%)	Vigor index I	Vigor index II
***Summer* maize**			
T_1_—*B. safensis* MF-01	86.25^b^	3987.57^d^	280.75^c^
T_2_—*B. altitudinis* MF-15	85.33^b^	4094.25^b^	294.10^b^
T_3_—*B. velezensis* MF-08	84.36^b^	4051.25^c^	300.25^b^
T_4_—*B. safensis* MF-01 + *B. altitudinis* MF-15 + *B. velezensis* MF-08	94.36^a^	4550.33^a^	405.92^a^
T_5_—Control (untreated)	76.50^c^	3280.43^e^	222.75^d^
***Kharif* maize**			
T_1_—*B. safensis* MF-01	86.33^b^	3806.15^d^	344.50^d^
T_2_—*B. altitudinis* MF-15	85.75^b^	3890.25^c^	351.85^c^
T_3_—*B. velezensis* MF-08	86.11^b^	3975.21^b^	360.01^b^
T_4_—*B. safensis* MF-01 + *B. altitudinis* MF-15 + *B. velezensis* MF-08	96.25^a^	4675.29^a^	444.50^a^
T_5_—Control (untreated)	76.20^c^	3042.25^e^	255.75^e^

*Data with different letters show significant difference in column data in randomized block design test at *p* < 0.05 under Duncan’s multiple range test.*

### Effects of Seed Biopriming and Microbial Inventerization on Root Architecture

**FIGURE 2:**
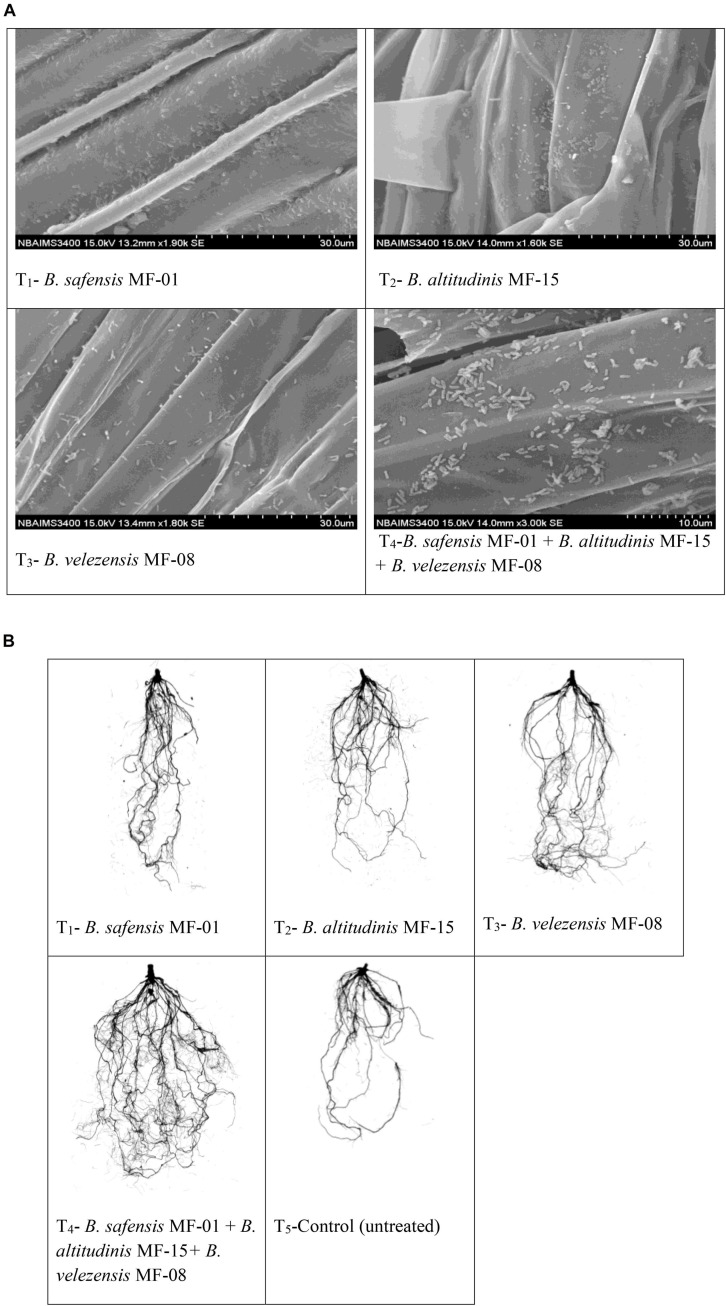
**(A)** Scanning electron microphotographs showing root colonization by selected strains of *Bacillus* spp. at 30 days of sowing. **(B)** Effects of seed bio-inoculation on root growth and development in the maize grown in saline sodic soil at 30 days of sowing. Treatments were as follows: T_1_—*B. safensis* MF-01, T_2_—*B. altitudinis* MF-15, T_3_—*B. velezensis* MF-08, T_4_—*B. safensis* MF-01 + *B. velezensis* MF-08 + *B. altitudinis* MF-15, and T_5_—control.

To see the effects of seed biopriming and microbial inventerization on root architecture, growth, and development, experiments were conducted in *Kharif* season under nethouse conditions. Scanning electron microphotographs clearly indicated that selected strains have the potential to colonize maize root even under saline–sodic soil at 30 DAS (Figure 2A). Upon inoculation and proper colonization of bioinoculants in the rhizosphere, an increased level of IAA was estimated (data not shown). Further, root scanning results clearly indicated that microbial inventerization has positive effects on root architecture and different parameters of root development. A significant (*p* ≤ 0.05) increase in the surface area (449.73 cm^2^), projected area (143.15 cm^2^), root length (2151.01 cm), length per volume (2151.01 cm/m^3^), total root volume (7.48 cm^3^), average diameter (0.75 mm), number of tips (4889.29), number of forks (7951.57), and number of crossings (2296.45) was recorded in the plant inoculated with all three strains, *B. safensis* MF-01, *B. altitudinis* MF-15, and *B. velezensis* MF-08 in combination compared to those inoculated with *B. safensis* MF-01, *B. altitudinis* MF-15, and *B. velezensis* MF-08 individually and untreated control under salt-stressed conditions. These values were as high as 2.5- to 3.5-fold over the untreated control plants (Table 3). However, significant differences in different parameters of the root growth and development were recorded in the plants inoculated with *B. safensis* MF-01, *B. altitudinis* MF-15, and *B. velezensis* MF-08 individually at 30 DAS (Table 3). The effects of seed biopriming with *B. safensis* MF-01, *B. altitudinis* MF-15, and *B. velezensis* MF-08 individually or in combination on enhancement of root growth and development were also evident from Figure 2B.

**TABLE 3:** Effects of seed biopriming and microbial intervention on root development and attributes in maize grown in *Kharif* season under nethouse experiments at 30 days of sowing.

Treatments	Surface area (cm^2^)	Projected area (cm^2^)	Root length (cm)	Length per volume (cm/m^3^)	Root volume (cm^3^)	Average diameter (mm)	Number of tips	Number of forks	Number of crossings
T_1_—*B. safensis* MF-01	174.46^d^	60.54^d^	1033.25^d^	1005.66^d^	2.04^b^	0.50^c^	2605.62^c^	4154.36^d^	595.25^d^
T_2_—*B. altitudinis* MF-15	190.21^c^	74.77^c^	1265.75^b^	1033.25^c^	2.12^b^	0.51^c^	2500.01^d^	4295.40^c^	805.26^c^
T_3_—*B. velezensis* MF-08	221.84^b^	78.57^b^	1138.28^c^	1138.82^b^	2.78^b^	0.54^b^	2727.50^b^	4410.25^b^	859.45^b^
T_4_—*B. safensis* MF-01 + *B. altitudinis* MF-15 + *B. velezensis* MF-08	449.73^a^	143.15^a^	2151.01^a^	2151.01^a^	7.48^a^	0.75^a^	4889.29^a^	7951.57^a^	2296.45^a^
T_5_—Control (untreated)	131.54^e^	41.87^e^	756.77^e^	756.77^e^	1.82^b^	0.48^d^	2019.25^e^	3021.45^e^	388.36^e^

*Data with different letters show significant difference in column data in randomized block design test at *p* < 0.05 under Duncan’s multiple range test.*

### Effect of Seed Biopriming and Microbial Inventerization on Expression of Genes Related to Root Development

**FIGURE 3:**
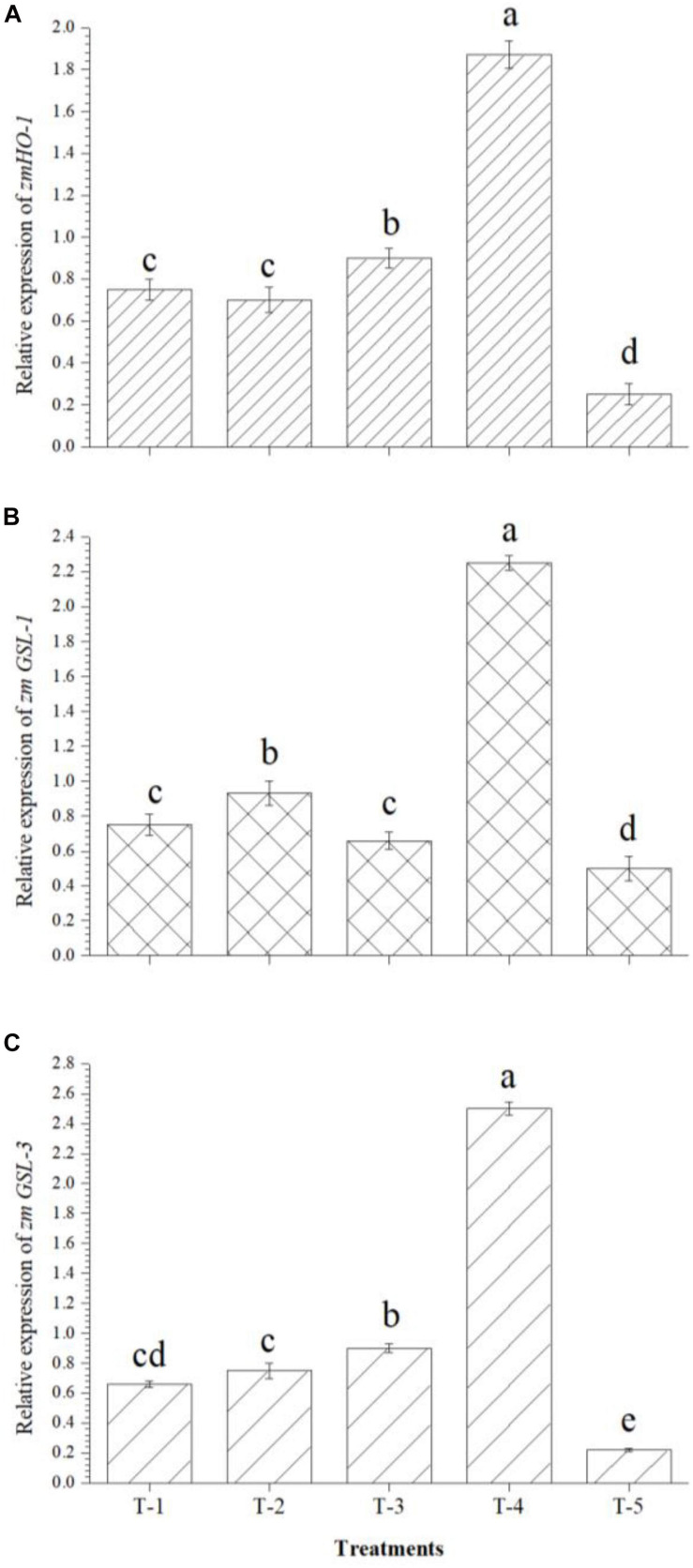
Effects of seed bio-inoculation on expression profile of genes (fold change) related to primary and secondary rooting in the maize roots grown in saline sodic soil at 30 days of sowing. **(A)** Relative expression of *zmHO-1*, **(B)** relative expression of *zmGSL-1*, and **(C)** relative expression of *zmGSL-3*. Treatments were as follows: T_1_—*B. safensis* MF-01, T_2_—*B. altitudinis* MF-15, T_3_—*B. velezensis* MF-08, T_4_—*B. safensis* MF-01 + *B. velezensis* MF-08 + *B. altitudinis* MF-15, and T_5_—control (untreated). Data are mean (*n* = 10) and vertical bar represents standard deviation. Data with different letters show significant difference in column data in randomized block design test at *p* < 0.05 under Duncan’s multiple range test.

After analyzing the root architecture data obtained from root scanning experiments, results prompted us to further investigate the up/down regulation of some of the important genes responsible for lateral root development in maize. Results indicated that *zmHO-1*, *zmGSL-1*, and *zmGSL-3* genes were up-regulated upon inoculation of *B. safensis* MF-01, *B. altitudinis* MF-15, and *B. velezensis* MF-08 individually or in combination in the maize roots even under saline–sodic soil at 30 DAS. It was observed that twofold increase in the expression of *zmHO-1* gene was recorded in the maize roots inoculated with the consortium of *B. safensis* MF-01, *B. altitudinis* MF-15, and *B. velezensis* MF-08 as compared to individual inoculation, whereas this value was as high as sevenfold over the untreated control plants (Figure 3A). Similarly, 2.75- to 3-fold increase in the expression of *zmGSL-1* (Figure 3B) and *zmGSL-3* (Figure 3C) was recorded in the maize roots inoculated with the *B. safensis* MF-01, *B. altitudinis* MF-15, and *B. velezensis* MF-08 in combination as compared to individually inoculated plants grown under saline–sodic soil. However, least expression of these genes was recorded in the untreated control plants. Results clearly indicated that these bioinoculants have the potential to modulate gene expression related to primary and secondary rooting in maize, which was also evidenced from root scanning results (Table 3).

### Effect of Seed Biopriming and Microbial Inventerization on Plant Growth Under Nethouse Conditions

Because of the significant differences observed in root architecture and growth parameters in response to seed biopriming in maize, nethouse experiments were conducted to evaluate the impact of selected strains on plant growth attributes under salt-stressed conditions. Results revealed that shoot length, root length, number of leaves, and fresh and dry weight of shoot and root were significantly increased by 2- to 2.5-fold in the plants bioprimed with consortium of all three strains *B. safensis* MF-01, *B. altitudinis* MF-15, and *B. velezensis* MF-08 as compared to untreated control plants in *Summer* maize grown in saline–sodic soil at 45 DAS (Table 4). In *Kharif* maize, shoot length, root length, number of leaves, and fresh weight of shoot were increased by ∼2-fold; however, fresh weight of root and dry weight of shoot and root were increased by ∼3-fold at 45 DAS under nethouse conditions. It was observed that individual application of either bioinoculant significantly enhanced the plant growth attributes tested by 1.25- to 1.5-fold as compared to untreated control plants in *Summer* and *Kharif* maize at 45 DAS under nethouse conditions (Table 4).

**TABLE 4:** Effects of seed biopriming and microbial intervention on plant growth parameters in maize grown in *Summer* and *Kharif* season under nethouse experiments at 45 days of sowing.

Treatments	Shoot length (cm)	Root length (cm)	Number of leaves plant^–1^	Fresh wt. of shoot (*g*)	Fresh wt. of root (g)	Dry wt. of shoot (g)	Dry wt. of root (g)
***Summer* maize**							
T_1_—*B. safensis* MF-01	27.33^c^	24.16^b^	4.06^c^	9.39^b^	3.66^c^	1.41^d^	1.25^c^
T_2_—*B. altitudinis* MF-15	27.66^c^	20.73^c^	4.33^c^	8.06^c^	4.09^c^	1.85^c^	1.33^c^
T_3_—*B. velezensis* MF-08	34.03^b^	25.06^b^	5.08^b^	10.66^b^	5.50^b^	2.05^b^	1.45^b^
T_4_—*B. safensis* MF-01 + *B. altitudinis* MF-15 + *B. velezensis* MF-08	45.35^a^	40.36^a^	7.48^a^	16.44^a^	8.33^a^	3.56^a^	2.50^a^
T_5_—Control (untreated)	24.26^d^	16.23^d^	3.15^d^	8.60^c^	3.05^d^	1.05^e^	0.95^d^
***Kharif* maize**							
T_1_—*B. safensis* MF-01	32.50^d^	28.46^b^	6.45^c^	10.25^b^	5.05^b^	1.62^c^	1.33^c^
T_2_—*B. altitudinis* MF-15	34.75^c^	25.55^c^	6.55^c^	10.45^b^	5.50^b^	1.95^b^	1.46^b^
T_3_—*B. velezensis* MF-08	36.46^b^	29.05^b^	7.25^b^	9.37^b^	5.33^b^	2.05^b^	1.55^b^
T_4_—*B. safensis* MF-01 + *B. altitudinis* MF-15 + *B. velezensis* MF-08	48.25^a^	42.33^a^	9.05^a^	15.44^a^	10.50^a^	4.75^a^	3.03^a^
T_5_—Control (untreated)	26.25^e^	20.23^d^	4.15^d^	7.05^c^	3.06^c^	1.25^d^	1.05^d^

*Data with different letters show significant difference in column data in randomized block design test at *p* < 0.05 under Duncan’s multiple range test.*

### Effect of Seed Biopriming and Microbial Inventerization on Accumulation of Stress-Responsive Biomolecules and Antioxidant Enzymes

**FIGURE 4:**
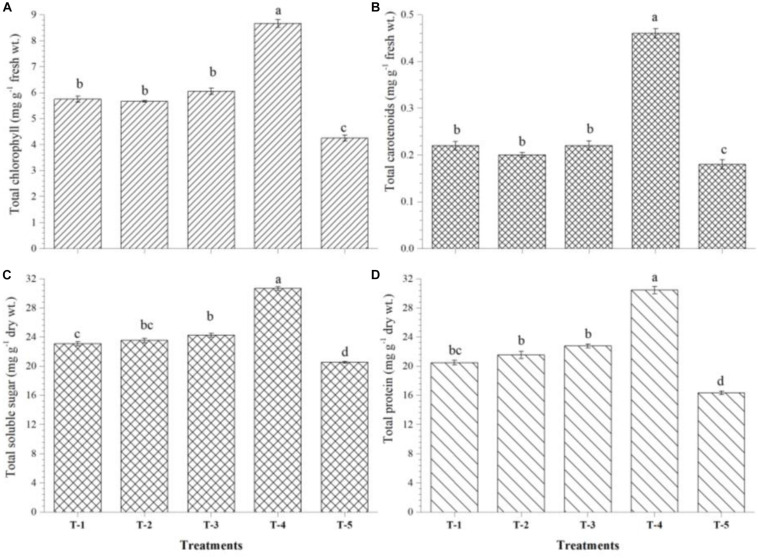
Effects of seed bio-inoculation on activities and accumulation of **(A)** total chlorophyll, **(B)** total carotenoids, **(C)** total soluble sugar, and **(D)** total protein in the maize leaves grown in saline sodic soil at 45 days of sowing. Treatments were as follows: T_1_—*B. safensis* MF-01, T_2_—*B. altitudinis* MF-15, T_3_—*B. velezensis* MF-08, T_4_—*B. safensis* MF-01 + *B. velezensis* MF-08 + *B. altitudinis* MF-15, and T_5_—control (untreated). Data are mean (*n* = 10) and vertical bar represents standard deviation. Data with different letters show significant difference in column data in randomized block design test at p < 0.05 under Duncan’s multiple range test.

Biosynthesis and accumulation of total chlorophyll, stress-responsive biomolecules, and antioxidant enzymes were significantly affected by seed biopriming and microbial inventerization in maize plants grown in saline–sodic soil. Results indicated that increased accumulation of total chlorophyll was recorded in the leaves of *Kharif* maize plants bioprimed with either bioagent (1.25- to 1.5-fold) or in combination of all three strains, i.e., *B. safensis* MF-01, *B. altitudinis* MF-15, and *B. velezensis* MF-08 (∼2-fold) as compared to untreated control plants (4.25 mg g^–1^ fresh wt.) at 45 DAS under nethouse conditions (Figure 4A). Similarly, total carotenoid content was increased by threefold in the leaves of *Kharif* maize plants bioprimed with consortium of all three strains (Figure 4B). Seed biopriming and microbial inventerization resulted in a significant increase in the total soluble sugar (Figure 4C) and total protein (Figure 4D) content in the leaves of *Kharif* maize plants treated with either of bioagent or in combination of all three relative to untreated control plants at 45 DAS under nethouse conditions.

**FIGURE 5:**
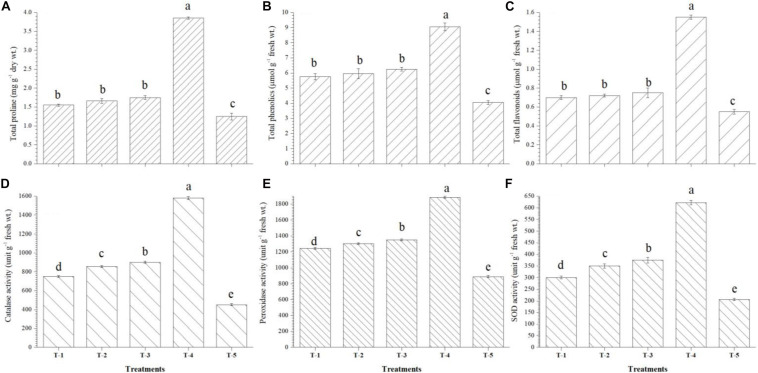
Effects of seed bio-inoculation on activities and accumulation of **(A)** total proline, **(B)** total phenolics, **(C)** total flavonoids, **(D)** catalase, **(E)** peroxidase, and **(F)** SOD in the maize leaves grown in saline sodic soil at 45 days of sowing. Treatments were as follows: T_1_—*B. safensis* MF-01, T_2_—*B. altitudinis* MF-15, T_3_—*B. velezensis* MF-08, T_4_—*B. safensis* MF-01 + *B. velezensis* MF-08 + *B. altitudinis* MF-15, and T_5_—control (untreated). Data are mean (*n* = 10) and vertical bar represents standard deviation. Data with different letters show significant difference in column data in randomized block design test at *p* < 0.05 under Duncan’s multiple range test.

It was also observed that maximum accumulation of total proline (3.85 mg g^–1^ dry wt.), total phenolics (9.05 μmol g^–1^ fresh wt.), and total flavonoids (1.55 μmol g^–1^ fresh wt.) was recorded in the leaves of *Kharif* maize plants bioprimed with consortium of all three strains, i.e., *B. safensis* MF-01, *B. altitudinis* MF-15, and *B. velezensis* MF-08 as compared to untreated control plants at 45 DAS under nethouse conditions. However, accumulation of total proline, total phenolics, and total flavonoids did not significantly differ in the leaves of maize plants bioprimed with either bioinoculant (Figures 5A–C). The data presented herein showed significant differences in the activity of catalase, peroxidase, and SOD recorded in the leaves of *Kharif* maize plants bioprimed with either strain and the consortium of all three strains. Significantly higher activity of catalase (1580.40 units *g*^–1^ fresh wt.), peroxidase (1885.45 units *g*^–1^ fresh wt.), and SOD (622.50 units *g*^–1^ fresh wt.) was recorded in the leaves of plants bioprimed with consortium of all three strains, i.e., *B. safensis* MF-01, *B. altitudinis* MF-15, and *B. velezensis* MF-08 followed by the plants bioprimed with *B. velezensis* MF-08, *B. altitudinis* MF-15, and *B. safensis* MF-01 individually at 45 DAS under nethouse conditions (Figures 5D–F). However, the lowest activity of catalase (452.25 units *g*^–1^ fresh wt.), peroxidase (890.25 units *g*^–1^ fresh wt.), and SOD (205.77 units *g*^–1^ fresh wt.) was recorded in the leaves of untreated control plants grown in saline–sodic soil under nethouse conditions at 45 DAS (Figures 5D–F).

### Effects on Expression of Stress-Responsive Genes and High-Affinity K^+^ Transporter

**FIGURE 6:**
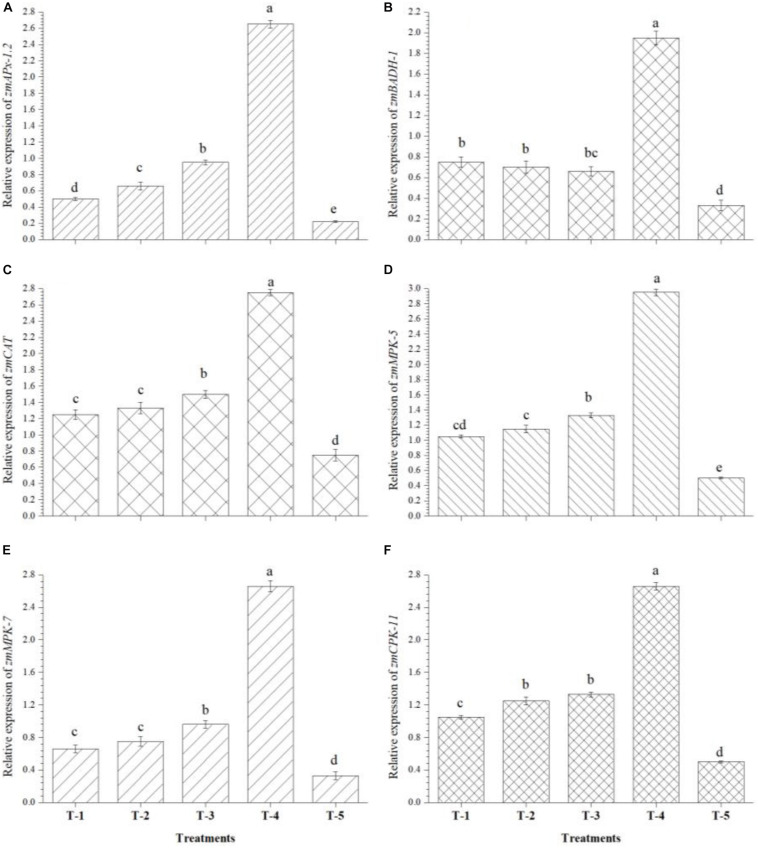
Effects of seed bio-inoculation on expression profile of genes (fold change) related to antioxidants and abiotic stress tolerance in the maize leaves grown in saline sodic soil at 45 days of sowing. **(A)** Relative expression of *zmAPx-1.2*, **(B)** relative expression of *zmBADH-1*, **(C)** relative expression of *zmCAT*, **(D)** relative expression of *zmMPK-5*, **(E)** relative expression of *zmMPK-7*, and **(F)** relative expression of *zmCPK-11*. Treatments were as follows: T_1_—*B. safensis* MF-01, T_2_—*B. altitudinis* MF-15, T_3_—*B. velezensis* MF-08, T_4_—*B. safensis* MF-01 *+ B. velezensis* MF-08 + *B. altitudinis* MF-15, and T_5_—control (untreated). Data are mean (*n* = 10) and vertical bar represents standard deviation. Data with different letters show significant difference in column data in randomized block design test at *p* < 0.05 under Duncan’s multiple range test.

Quantitative real time-PCR analysis was carried out to study the effects of seed biopriming and microbial inventerization on the regulation of genes related to antioxidants and MAP kinase in the leaves of *Kharif* maize grown in saline–sodic soil under nethouse conditions at 45 DAS. Interestingly, seed biopriming with either of the strains or a consortium of all three strains, i.e., *B. safensis* MF-01, *B. altitudinis* MF-15, and *B. velezensis* MF-08 under salt stress significantly induced the expression levels of *zmAPx-1.2*, *zmBADH-1*, *zmCAT*, *zmMPK-5*, *zmMPK-7*, and *zmCPK-11* genes in leaf tissues as compared to the untreated control plants (Figures 6A–F). The results indicated that an increased expression was recorded in *zmAPx-1.2* by 12-fold, *zmBADH-1* by 5-fold, *zmCAT* by 3.5-fold, *zmMPK-5* by 4-fold, *zmMPK-7* by 8-fold, and *zmCPK-11* by 5-fold genes in the leaves of plants bioprimed with consortium of all three strains compared to untreated control. Additionally, differential expression of these genes was also observed in the plants bioprimed with either of the bioinoculants individually, while untreated control plants showed the lowest expression of all these genes at 45 DAS under nethouse conditions (Figures 6A–F).

**FIGURE 7:**
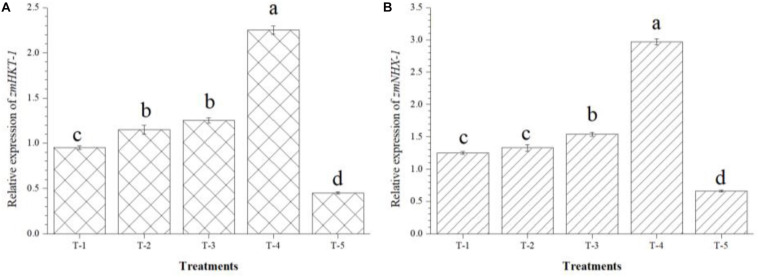
Effects of seed bio-inoculation on expression profile of **(A)** *zmHKT-1* and **(B)** *zmNHX1* genes (fold change) in the maize roots grown in saline sodic soil at 45 days of sowing. Treatments were as follows: T_1_—*B. safensis* MF-01, T_2_—*B. altitudinis* MF-15, T_3_—*B. velezensis* MF-08, T_4_—*B. safensis* MF-01 *+ B. velezensis* MF-08 + *B. altitudinis* MF-15, and T_5_—control (untreated). Data are mean (*n* = 5) and vertical bar represents standard deviation. Data with different letters show significant difference in column data in randomized block design test at *p* < 0.05 under Duncan’s multiple range test.

Further, the possible role of *B. safensis* MF-01, *B. altitudinis* MF-15, and *B. velezensis* MF-08 in modulating the expression of gene(s) involved in the abiotic stress tolerance especially salt was further investigated by analyzing the expression profile of *zmHKT1* and *zmNHX1* in maize roots under salt-stressed conditions. In addition, at least *zmHKT1* and *zmNHX1* genes were identified as being up-regulated under salt-stressed conditions. qRT-PCR results revealed that 2- to 2.25-fold increase in the transcript level of *zmHKT1* and *zmNHX1* was recorded in the maize roots inoculated with the *B. safensis* MF-01, *B. altitudinis* MF-15, and *B. velezensis* MF-08 in combination as compared to the maize plants inoculated with individual strain. However, the lowest expression of *zmHKT1* and *zmNHX1* was recorded in the roots of uninoculated control plants (0.45 and 0.66, respectively), emphasizing the role of bioinoculants in modulating gene expression and strengthening the physio-biochemical homeostasis that leads to salt tolerance in maize grown in saline–sodic soil at 45 DAS (Figure 7).

### Effect of Seed Biopriming and Microbial Inventerization on Soil Enzymes, MBC, and Soil Respiration

Seed biopriming and microbial inventerization led to differential activation of soil enzymes in the rhizosphere of *Kharif* maize grown in saline–sodic soil under nethouse conditions. Maximum dehydrogenase activity was recorded in the rhizosphere of plants bioprimed with consortium of all three strains (28.56 μg TPF/g of dry soil/24 h) as compared to untreated control (18.93 μg TPF/g of dry soil/24 h). However, dehydrogenase activity did not differ significantly in the rhizosphere of *Kharif* maize bioprimed with either of the bioinoculants (Table 5). The alkaline and acid phosphatase activity was found to be significantly higher in rhizosphere soil of *Kharif* maize bioprimed with consortium of all three strains (124.50 and 115.85 μg PNP/g of dry soil/2 h, respectively). A more or less similar pattern was recorded for protease, cellulose, and invertase activity in the rhizosphere of maize bioprimed with consortium of all three microbial strains. On the other hand, the increment in the activity of soil enzymes was also significantly higher in the rhizosphere of maize plants bioprimed with individual microbial strains as compared to untreated control at 45 DAS (Table 5).

**TABLE 5:** Effects of seed biopriming and microbial intervention on activity of soil enzymes in maize rhizosphere grown in *Kharif* season under nethouse experiments at 45 days of sowing.

Treatments	Dehydrogenase activity (μg TPF/g of dry soil/24 h)	Alk. phosphatase activity (μg PNP/g of dry soil/2 h)	Acid phosphatase activity (μg PNP/g of dry soil/2 h)	Protease activity (μg tyrosine/g of dry soil/h)	Cellulase activity (μg glucose/g of dry soil/h)	Invertase activity (mg glucose/g of dry soil/h)	Microbial biomass carbon (mg/kg of dry soil)	Soil respiration (mg CO_2_-C/100 *g* of dry soil/10 day)
T_1_—*B. safensis* MF-01	20.33^b^	101.50^b^	94.33^bc^	105.50^c^	70.50^d^	5.25^c^	114.56^c^	7.85^c^
T_2_—*B. altitudinis* MF-15	20.80^b^	98.05^c^	95.85^b^	110.50^b^	73.57^c^	6.05^b^	112.75^d^	8.01^c^
T_3_—*B. velezensis* MF-08	20.50^b^	101.95^b^	96.33^b^	109.75^b^	75.50^b^	6.45^b^	116.25^b^	9.33^b^
T_4_—*B. safensis* MF-01 + *B. altitudinis* MF-15 + *B. velezensis* MF-08	28.5^a^	124.50^a^	115.85^a^	119.50^a^	88.85^a^	10.01^a^	143.60^a^	14.59^a^
T_5_—Control (untreated)	18.93^c^	88.45^d^	83.25^d^	98.25^d^	60.33^e^	4.35^d^	111.25^de^	6.50^d^

*Data with different letters show significant difference in column data in randomized block design test at *p* < 0.05 under Duncan’s multiple range test.*

### Effect of Seed Biopriming and Microbial Inventerization on Under Field Conditions

Looking at the significant impact of biopriming and microbial inventerization on seed germination, vigor indices, root architectural development, plant growth, activity of soil enzymes, and physio-biochemical modulation in maize grown in saline–sodic soil under nethouse experiments, field experiments are conducted to evaluate the impact of these strains on plant growth attributes, physiological traits, yield, and yield-attributing parameters and uptake of Na^+^, K^+^, and Ca^2+^ in maize plants grown in saline–sodic soil.

### Effects on Plant Growth and Physiological Traits

Plants grown under saline–sodic soil and seed biopriming with *B. safensis* MF-01, *B. altitudinis* MF-15, and *B. velezensis* MF-08 individually or in combination of all three strains significantly enhance the plant height in *Summer* and *Kharif* maize grown under field conditions. The maximum plant height was recorded in the *Summer* and *Kharif* maize bioprimed with consortium of all three strains (175.66 and 206.75 cm, respectively) followed by *B. velezensis* MF-08 (140.25 and 148.96 cm, respectively) and *B. altitudinis* MF-15 (132.33 and 142.33 cm, respectively) individually inoculated plants (Table 6). The minimum plant height was recorded in the untreated control plants during the *Summer* and *Kharif* season (120.23 and 120.25 cm, respectively). Similarly, maximum number of leaves and dry weight of shoot and root were recorded in the *Summer* (17.25, 42.33 *g*, and 22.66 *g*, respectively) and *Kharif* (10.23, 62.46 *g*, and 36.40 *g*, respectively) maize plants bioprimed with consortium of all three strains. However, a slight difference in the number of leaves and dry weight of shoot and root was recorded in the *Summer* and *Kharif* maize plants bioprimed with either strain but significantly higher than untreated control plants at 45 DAS. Further in *Kharif* maize, all growth parameters were significantly higher when compared with the *Summer* maize under salt-stressed conditions (Table 6).

**TABLE 6:** Effects of seed biopriming and microbial intervention on plant growth parameters in maize grown in *Summer* and *Kharif* season under field conditions at 45 days of sowing.

Treatments	Plant height (cm)	Number of leaf	Dry wt. of shoot (g)	Dry wt. of root (g)
***Summer* maize**				
T_1_—*B. safensis* MF-01	125.51^d^	12.66^b^	29.66^b^	14.35^c^
T_2_—*B. altitudinis* MF-15	132.33^c^	13.02^b^	28.25^b^	15.03^c^
T_3_—*B. velezensis* MF-08	140.25^b^	13.66^b^	30.50^b^	16.46^b^
T_4_—*B. safensis* MF-01 + *B. altitudinis* MF-15 + *B. velezensis* MF-08	175.66^a^	17.25^a^	42.33^a^	22.66^a^
T_5_—Control (untreated)	120.23^e^	9.75^c^	25.67^c^	11.42^d^
***Kharif* maize**				
T_1_—*B. safensis* MF-01	137.45^d^	6.96^b^	31.96^c^	18.33^c^
T_2_—*B. altitudinis* MF-15	142.33^c^	7.25^b^	32.33^c^	20.65^b^
T_3_—*B. velezensis* MF-08	148.96^b^	7.06^b^	36.44^b^	20.25^b^
T_4_—*B. safensis* MF-01 + *B. altitudinis* MF-15 + *B. velezensis* MF-08	206.75^a^	10.23^a^	62.46^a^	36.40^a^
T_5_—Control (untreated)	120.25^e^	6.06^c^	26.45^d^	15.25^d^

*Data with different letters show significant difference in column data in randomized block design test at *p* < 0.05 under Duncan’s multiple range test.*

It was clearly indicated that seed biopriming and microbial inventerization have a positive impact on plant growth and physiological traits of *Summer* and *Kharif* maize grown in saline–sodic soil. According to data obtained and analyzed, maximum leaf area index, mean crop growth rate, mean relative growth rate, and mean net assimilation rate were recorded in the plants bioprimed with consortium as compared to individual inoculation and untreated control plants in both *Summer* (2.66, 15.10, 15.75, and 5.86, respectively) and *Kharif* (3.50, 20.45, 21.76, and 8.02, respectively) maize at 45 DAS (Table 7). However, slight deviation in the leaf area index, mean crop growth rate, mean relative growth rate, and mean net assimilation rate was recorded in the *Summer* and *Kharif* maize plants bioprimed with either strain, but these values were significantly higher than those of the untreated control plant at 45 DAS (Table 7). Therefore, these strains were able to promote plant growth and physiological traits of *Summer* and *Kharif* maize grown under salt-stressed conditions. It was concluded that the highest growth attributes were recorded in the plants treated with consortium. In general, among the individual inoculation, the highest growth attributes were recorded with *B. velezensis* MF-08.

**TABLE 7:** Effects of seed biopriming and microbial intervention on physiological traits in maize grown in *Summer* and *Kharif* season under field conditions at 45 days of sowing.

Treatments	Leaf area index	Mean crop growth rate (g/m^2^/day)	Mean relative growth rate (mg/g/day)	Mean net assimilation rate (g/m^2^ leaf area/day)
***Summer* maize**				
T_1_—*B. safensis* MF-01	2.05^b^	11.27^c^	13.50^b^	4.50^b^
T_2_—*B. altitudinis* MF-15	2.15^b^	12.50^b^	13.75^b^	4.45^b^
T_3_—*B. velezensis* MF-08	2.25^b^	12.66^b^	13.05^c^	4.25^bc^
T_4_—*B. safensis* MF-01 + *B. altitudinis* MF-15 + *B. velezensis* MF-08	2.66^a^	15.10^a^	15.75^a^	5.86^a^
T_5_—Control (untreated)	1.86^bc^	10.05^d^	12.47^d^	3.85^d^
***Kharif* maize**				
T_1_—*B. safensis* MF-01	2.75^c^	14.35^c^	17.25^b^	5.79^c^
T_2_—*B. altitudinis* MF-15	2.81^b^	15.06^b^	18.46^c^	5.85^c^
T_3_—*B. velezensis* MF-08	2.89^b^	15.33^b^	17.75^b^	6.05^b^
T_4_—*B. safensis* MF-01 + *B. altitudinis* MF-15 + *B. velezensis* MF-08	3.50^a^	20.45^a^	21.76^a^	8.02^a^
T_5_—Control (untreated)	2.55^d^	12.46^d^	15.67^d^	5.25^d^

*Data with different letters show significant difference in column data in randomized block design test at *p* < 0.05 under Duncan’s multiple range test.*

### Effects on Yield and Yield-Attributing Traits

Seed biopriming and microbial inventerization significantly affect the yield-attributing traits (number of cobs/plant, cob size, number of grain/cob, and test weight) and grain yield of *Summer* and *Kharif* maize grown in saline–sodic soil. The maximum number of cobs/plant, cob size, number of grain/cob, test weight, and grain yield was recorded in the *Summer* (3.50, 18.46 cm, 314.25, 344.75 *g*, and 24.56 kg, respectively) and *Kharif* (3.15, 29.45 cm, 385.44, 365.33 *g*, and 33.46 kg, respectively) maize plants bioprimed with consortium of all three strains (Table 8). In individually inoculated plants, a slight deviation was recorded in the number of cobs/plant, cob size, number of grain/cob, test weight, and grain yield in the maize grown in salt-stressed conditions, but these values were significantly higher as compared to un-inoculated control (Table 8). However, minimum grain yield was reported in the untreated control plants in both seasons (15.33 and 24.81 kg, respectively). The data presented herein showed significant differences in the yield-attributing traits and grain yield between *Summer* and *Kharif* maize across the treatments under salt-stressed conditions. In conclusion, the highest grain yield was recorded in *Kharif* maize as compared to the *Summer* maize bioprimed with consortium. Among the individual inoculation strains, MF-08 bioprimed plants gave maximum yield in both seasons.

**TABLE 8:** Effects of seed biopriming and microbial intervention on yield and yield-attributing traits in maize grown in *Summer* and *Kharif* season under field conditions at harvest.

Treatments	Number of cobs/plant	Cob size (cm)	Number of grain/cob	Test weight (*g*)	Yield/plot (kg)
***Summer* maize**					
T_1_—*B. safensis* MF-01	1.80^b^	11.85^b^	240.57^d^	288.47^b^	18.66^b^
T_2_—*B. altitudinis* MF-15	2.05^b^	12.75^b^	250.47^c^	290.37^b^	19.01^b^
T_3_—*B. velezensis* MF-08	2.15^b^	12.45^b^	260.45^b^	285.49^c^	19.55^b^
T_4_—*B. safensis* MF-01 + *B. altitudinis* MF-15 + *B. velezensis* MF-08	3.05^a^	18.46^a^	314.25^a^	344.57^a^	24.56^a^
T_5_—Control (untreated)	2.00^b^	10.15^c^	225.45^e^	275.75^d^	15.23^c^
***Kharif* maize**					
T_1_—*B. safensis* MF-01	2.25^b^	14.75^b^	309.25^b^	298.23^c^	28.10^b^
T_2_—*B. altitudinis* MF-15	2.30^b^	14.96^b^	290.75^d^	300.97^c^	28.50^b^
T_3_—*B. velezensis* MF-08	2.50^b^	15.25^b^	300.25^c^	315.46^b^	28.01^b^
T_4_—*B. safensis* MF-01 + *B. altitudinis* MF-15 + *B. velezensis* MF-08	3.15^a^	29.45^a^	385.44^a^	365.33^a^	33.46^a^
T_5_—Control (untreated)	1.95^b^	12.47^c^	254.33^e^	285.25^d^	24.81^c^

*Data with different letters show significant difference in column data in randomized block design test at *p* < 0.05 under Duncan’s multiple range test.*

### Effects on Uptake of Na^+^, K^+^, and Ca^2+^

To see the effects of seed biopriming and microbial inventerization on the uptake and translocation of Na^+^, K^+^, and Ca^2+^ in maize, an analysis was done to estimate the uptake and translocation of these ions in the *Kharif* maize grown in saline–sodic soil under field experiments. Under salt stress conditions, Na^+^ content in the maize roots was significantly higher in the untreated control plants (26.75) as compared to the roots of other treated plants. The least Na^+^ was recorded in the roots of maize plants bioprimed with consortium of all three strains, i.e., *B. safensis* MF-01, *B. altitudinis* MF-15, and *B. velezensis* MF-08 (7.25) followed by *B. velezensis* MF-08 (18.01), *B. altitudinis* MF-15, (19.25), and *B. safensis* MF-01 (20.47) inoculated plants. In contrast, maximum amounts of K^+^ (45.37) and Ca^2+^ (20.33) were recorded in the roots of *Kharif* maize plants inoculated with consortium of all three strains. However, plants inoculated with either strain had a smaller deviation (in general, non-significant) in K^+^ and Ca^2+^ content in the roots of maize plants (Table 9). Taken together, there was a ∼13-fold increment in Na^+^/K^+^ ratio for roots of untreated plants (2.18) and those inoculated with the consortium of all three strains (0.16).

**TABLE 9:** Effects of seed biopriming and microbial intervention on Na^+^, K^+^, and Ca^2+^ uptake in maize grown in *Kharif* season under field conditions at harvest.

Treatments	Na^+^	K^+^	Ca^2+^	Na^+^/K^+^
**Content in root**				
T_1_—*B. safensis* MF-01	20.47^b^	20.33^b^	12.66^b^	1.01^b^
T_2_—*B. altitudinis* MF-15	19.25^b^	20.25^b^	12.75^b^	0.95^b^
T_3_—*B. velezensis* MF-08	18.01^b^	22.35^b^	12.50^b^	0.81^b^
T_4_—*B. safensis* MF-01 + *B. altitudinis* MF-15 + *B. velezensis* MF-08	7.25^c^	45.37^a^	20.33^a^	0.16^c^
T_5_—Control (untreated)	26.75^a^	12.25^c^	11.50^c^	2.18^a^
**Content in shoot**				
T_1_—*B. safensis* MF-01	13.06^b^	8.25^bc^	9.25^b^	1.58^b^
T_2_—*B. altitudinis* MF-15	12.96^b^	9.75^b^	9.33^b^	1.33^c^
T_3_—*B. velezensis* MF-08	12.47^b^	10.50^b^	9.56^b^	1.19^c^
T_4_—*B. safensis* MF-01 + *B. altitudinis* MF-15 + *B. velezensis* MF-08	5.25^c^	24.75^a^	16.05^a^	0.21^d^
T_5_—Control (untreated)	15.75^a^	5.67^d^	7.50^c^	2.78^a^

*Data with different letters show significant difference in column data in randomized block design test at *p* < 0.05 under Duncan’s multiple range test.*

Further, results showed that shoots of untreated plants had a Na^+^ content nearly threefold higher (15.75) than plants bioprimed with consortium of all three strains (5.25), whereas plants bioprimed with either microbial strain had comparatively less Na^+^ content in the shoot than the untreated control plants. Similar to the root K^+^ and Ca^2+^, maximum level of K^+^ and Ca^2+^ was reported from the shoot of maize plants bioprimed with consortium of microbial strains tested (24.75 and 16.05, respectively). No significant differences were recorded in the K^+^ and Ca^2+^ content in the shoot of maize plants bioprimed with either of the microbial strains at harvest (Table 9). However, the least amount of K^+^ and Ca^2+^ was reported from the shoot of untreated maize plants grown in the field under salt-stressed conditions (5.67 and 7.50, respectively). The results implied that strains *B. safensis* MF-01, *B. altitudinis* MF-15, and *B. velezensis* MF-08 reduced the uptake of Na^+^ and increased the uptake of K^+^ and Ca^2+^ and thus help in maintaining the ion balance and, thus, increase salt tolerance in bioprimed maize.

## Discussion

Salt stress is one of the important abiotic stresses affecting crop establishment, survival, and productivity of maize, leading to severe economic losses year after year across the globe (Singh S. et al., 2019). Plant breeders routinely explore the wild relative, which may have prominent gene(s)/QTLs for salt stress tolerance and conduct multi-location trials over years to evaluate the performance of test entries against salt stress (Niu et al., 2012; Farooq et al., 2015; Singh S. et al., 2019). Alternatively, plant-/rhizo-microbiome plays an important role and their interactions in the rhizosphere influence plant fitness and improved functioning of ecosystems under salt-stressed conditions (Bais et al., 2006; Hameeda et al., 2008; Irfan et al., 2019). This may be accomplished through several direct and indirect mechanisms including hormone-induced growth stimulation (Zong et al., 2009), signaling (Zhu, 2002), chemotaxis, and modulation of physio-biochemical pathways in the plant system (Zhu, 2003; Zahra et al., 2018). The aim of the present study was to elucidate the microbe-mediated physio-biochemical and molecular mechanisms that provide salt tolerance, ecological fitness, and adaptiveness to maize plants grown in saline–sodic soil. In the current study, we have focused on the cooperative interactions of native *Bacillus* spp. with host defense responses underlying activation of stress tolerance cascades in maize plants grown in saline–sodic soil. This study provides some of the unique findings with a mechanistic overview of microbe-mediated stress tolerance in maize. Results of this investigation clearly indicated that maize rhizosphere harbors rich diversity of *Bacillus* spp. Results show that 20 different species of *Bacillus* were isolated and characterized from maize rhizosphere. These isolates have the potential to tolerate high concentrations of NaCl and have good PGP traits. They solubilize phosphorus, potassium, and zinc, and make them available to crop plants and promote plant growth directly and/or indirectly under salt-stressed conditions. Moreover, several reports indicated that rhizosphere microorganisms play an important role in alleviating salt stress in many crop plants in a different manner (Saleem et al., 2018; Rouphael et al., 2020; Wang et al., 2020). *Bacillus* spp. are a root-associated mutualistic plant symbiont with a capability to colonize roots, nourish the host, and protect plants from biotic and abiotic stresses via developing biofilm on the host root system (Ahmad et al., 2008; Hameeda et al., 2008; Singh et al., 2016a). Results clearly indicated that test strains, *B. safensis* MF-01, *B. altitudinis* MF-15, and *B. velezensis* MF-08 profusely colonized the maize root inoculated individually or in consortium and formed a thin biofilm on the root system (Figure 2A). Host species, plant secretome, and microenvironment of a niche are the key deciding factors in the recruitment and shaping of a rhizosphere microbiome (Bais et al., 2006; Micallef et al., 2009; Mahoney et al., 2017). These microorganisms formed biofilm on the root and established a close relation with their host system and thereby stimulate plant vigor under biotic and abiotic stresses (Sahu et al., 2019; Singh S. et al., 2019; Singh U. B. et al., 2019; Singh et al., 2020). However, interactions between host and rhizosphere microorganisms may change due to the increasing global temperature, salt, and environmental factors (Liu et al., 2006; Souza et al., 2015; Szoboszlay et al., 2015). In the changing climatic scenario and constant increase in the area under salinity and/or alkalinity, these microbial inoculants play a key role in the establishment of young seedlings under salt-stressed conditions. This phenomenon is called IST (Bais et al., 2006; Singh et al., 2016a; Singh U. B. et al., 2019).

It is necessary to explore and characterize microbial strain(s) and to develop effcient, stable, and eco-friendly bioformulations that can be utilized to protect plants from toxic effects of salt stress. In the present study, liquid-based bioformulations have been developed using a unique medium that supports the bacterial population (log colony-forming units) for a longer time (data not shown). For a long time, biological preparations from spore-forming *Bacillus* spp. were preferred and used as efficient microbial inoculants successfully for the management of salt stress in many crops (Liu et al., 2006; Singh et al., 2016a). Because of their wider adoptability, positive rhizosphere effects, and long-term viability (shelf life), they facilitate the development of successful commercial products (Singh et al., 2016a; Bokhari et al., 2019; Sahu et al., 2019). They have the potential to colonize and spread in the root, rhizosphere soil, and foliar environments. Simultaneously, they are capable of suppressing growth of harmful biotic entities and/or ill effects of abiotic stresses effectively (Liu et al., 2006; Singh et al., 2016a; Sahu et al., 2019). Results indicated that seed biopriming and microbial inventerization have positive effects on root architecture and root development. Many fold increases in the surface area, projected area, root length, length per volume, total root volume, average diameter, number of tips, number of forks, and number of crossings were reported in the plants inoculated with either of bioagents or in combination compared to untreated control under salt-stressed conditions. Indeed, lateral roots are critical to plant anchorage, nutrient acquisition, and water uptake. Development of secondary and tertiary roots (lateral roots) of a plant is greatly influenced under salt stress conditions. It was shown that seed biopriming and microbial inventerization modulate the expression of several genes (*ZmHO-1*, *ZmGSL-1*, and *ZmGSL-3*) involved in lateral root development. Further, auxin (IAA) synthesized by the microbial inoculants in the rhizosphere soil might be involved in the modulation of cascades responsible for root architecture development (Berberich et al., 1999; Du and Scheres, 2018). Moreover, lateral root formation is a highly complex process that involves in general three major stages such as lateral root initiation, formation of the lateral root primordia, and finally lateral root emergence (Lalle et al., 2005; Kashyap et al., 2018; He and Meng, 2020). Several pathways/cascades were investigated in model plants including *Arabidopsis thaliana* in which plant growth regulators, auxins, play an important role at multiple stages of the lateral root development (Lalle et al., 2005; Lynch and Brown, 2012; Santos Teixeira and Ten Tusscher, 2019). The auxin gradient and signaling determine cell fate during lateral root primordial development while, during emergence, auxin regulates cell separation in the roots (Tian et al., 2014). Further, a small amount of auxin activates the MKK4/MKK5–MPK3/MPK6 cascade via TMK1/TMK4, which regulates the cell division pattern during lateral root morphogenesis (Zhu et al., 2019; Xu et al., 2020). The increased expression of *ZmHO-1*, *ZmGSL-1*, and *ZmGSL-3* genes and secondary and tertiary roots in the bioprimed plants provides a proof of concept that bioinoculant(s) might modulate the physio-biochemical pathways at the molecular level, which could potentially lead to lateral root development and substantially increase the establishment of maize plants even under salt-stressed conditions (Zimmermann et al., 2010; Zhang et al., 2018). However, the exact role of microbial inoculants in the activation of MAPK signaling during lateral root formation is not yet fully elucidated and needs further in-depth investigation.

Moreover, high salt concentration in the soil solutes causes ionic imbalance and osmotic stress in the root system. Later on, excess Na^+^ inhibits/alters cellular metabolism, photosynthesis, and protein and lipid biosynthesis, thereby limiting the root growth and ultimately leading to early seedling mortality (Cicek and Çakirlar, 2002; Chaves et al., 2009; Niu et al., 2012; Delavar et al., 2018; Cao et al., 2020). Additionally, excess Na^+^ in the soil solute had negative effects on plant growth, soil microbial activity, and uptake and translocation of essential nutrients in the plants (Singh S. et al., 2019). In the present study, a dramatic decline was reported in plant growth parameters such as plant height, shoot and root length, number and size of leaf, and fresh and dry weight in untreated *Summer* and *Kharif* maize grown in saline–sodic soil, possibly due to higher accumulation and toxicity of Na^+^ and osmotic stress caused by impaired ion homeostasis and hampered overall growth performance (Zhu, 2003; Zhang et al., 2006, 2019). However, under salt-stressed conditions, *Summer* and *Kharif* maize bioprimed with either of the strains or in consortium of all three significantly enhanced plant growth and fresh and dry biomass accumulation compared to untreated control under nethouse and field conditions. These results were in agreement with previous work, which showed that rhizospheric as well as endophytic microbes increased shoot and root growth of several agriculturally important crops like rice (Jeong et al., 2006), maize (Singh U. B. et al., 2019), wheat (Singh et al., 2016a), and tomato (Vaishnav et al., 2020) under salt stress conditions. This might be due to the increased availability of essential nutrients, ionic balance in the soil solutes, and reduced osmotic stress in the plants. Further, several plant-associated beneficial microorganisms produce plant growth regulators, small peptides, and signaling molecules in the rhizosphere that would directly encourage/regulate plant growth or modulate certain pathways directly and/or indirectly under different circumstances (Singh et al., 2016a, b; Singh S. et al., 2019; Singh U. B. et al., 2019). In the present study, a drastic decline in leaf chlorophyll was recorded in the untreated control plants as compared to bioprimed plants grown in saline–sodic soil. Seed biopriming with compatible microbe(s) was found to improve chlorophyll content under salt stress conditions, which ultimately enhanced the photosynthesis and significantly increased the level of total soluble sugar and protein recorded. The drastic reduction in chlorophyll and other pigments might be due to inefficient activities of the enzymes α-aminolevulinic acid dehydratase and proto chlorophyllide reductase, which are coordinately involved in biosynthesis of chlorophyll and other pigments. Our results are in agreement with Latef and Tran (2016) who reported that Na^+^ toxicity reduced chlorophyll contents in maize plants subjected to salt stress.

The inherent redox nature of salts, specifically sodium salts, accelerates toxicity by generating reactive oxygen species (ROS), such as hydroxyl radicals (^⋅^OH), hydrogen peroxide (H_2_O_2_), and superoxide anions (O_2_^⋅–^) in the plant system (Liu et al., 2012, 2013; Meng et al., 2016). To conquer salt-induced oxidative stress, plant cells have a well-developed inherent antioxidant capability that is composed of non-enzymatic components, such as glycine betaine, proline, trehalose, ascorbic acid, glutathione, and other organic osmolytes and enzymatic components, such as catalase, peroxidase, SOD, glutathione peroxidase, ascorbate peroxidase, dehydroascorbate reductase, glutathione *S-*transferase, glutathione reductase, monodehydroascorbate reductase, etc. (Pan et al., 2012; Jiang et al., 2016; Phillips et al., 2018; Nawaz and Wang, 2020). Moreover, high salt stress causes cytotoxicity to cellular biomolecules. More specifically, it causes brutal oxidative damage to proteins and nucleic acids either (i) directly through accelerating ROS production, or (ii) indirectly by the overproduction of advanced glycation end products, which leads to membrane disruption, exo-osmosis, and cell death (Jiang et al., 2016; Phillips et al., 2018). In this study, significantly higher accumulation of compatible osmolyte proline (∼3.5-fold), phenolics (∼2.5-fold), and flavonoids (∼3.0-fold) has been reported in the plants bioprimed with consortium of compatible strains *B. safensis* MF-01, *B. altitudinis* MF-15, and *B. velezensis* MF-08 grown in saline–sodic soil. They are a noble indicator of salt stress tolerance (Sakamoto and Murata, 2002; Wu et al., 2011). Likewise, several-fold increment in the activity of catalase, peroxidase, and SOD was reported in the plants bioprimed with either of the strains or in combination of all three strains. The present result is supported by several researchers who reported that activity of these antioxidant enzymes in maize under salt stress has been significantly increased by the application of compatible microbial inoculants (Mittler et al., 2004; Singh U. B. et al., 2019). Further, *zmAPx-1.2*, *zmBADH-1*, *zmCAT*, *ZmMPK5*, *ZmMPK7*, and *ZmCPK11* were markedly up-regulated in the plants bioprimed with microbial inoculants individually or in combination, expected to contribute in the improvement of antioxidant defense systems under salt-stressed conditions (Zhang et al., 2006; Yamane et al., 2010; Jiang et al., 2016; Phillips et al., 2018). It was reported that *ZmMPK5* is the key gene activated by H_2_O_2_ and regulates the antioxidant defense systems, while *ZmCPK11* increases the activity and expression of antioxidant enzymes such as SOD and ascorbate peroxidase under stressed conditions (Zhang et al., 2010; Ding et al., 2013). Moreover, increased activation of *ZmMPK7* was reported during osmotic stress for alleviation of toxic effects of reactive oxygen species (ROS) and SA-regulated broad-spectrum resistance to biotic stresses, thereby regulating plant growth and development (Zong et al., 2009; Shi et al., 2010).

It is well established that Na^+^/K^+^ homeostasis and low cytosolic Na^+^ concentrations are the key factors for salt tolerance in plants (Jiang et al., 2016; Singh et al., 2016a; Singh S. et al., 2019). Generally, under salt-stressed conditions, comparatively higher uptake and bioaccumulation of Na^+^ inside the plant cells inhibit K^+^ uptake, resulting in an increase in Na^+^/K^+^ ratio that adversely affects plant growth and development (Jiang et al., 2016). In the present study, a significant decrease in Na^+^ content and an increase K^+^ and Ca^2+^ were recorded in the root and shoot of the maize plants bioprimed with all three strains individually and in consortium. It was reported that microbial inoculants may regulate the uptake of Na^+^, K^+^, and Ca^2+^ and maintain ionic homeostasis/equilibrium in the plants directly and/or indirectly. These results were in agreement with the previous report where plants bioprimed with endophytic *P. geniculata* MF-84 showed significantly high K^+^ and Ca^2+^ concentrations in the plants as compared to untreated control that resulted in enhancement of salt tolerance (Singh S. et al., 2019). In maize roots, significantly high expression of Na^+^ and K^+^ transporter genes such as *zmNHX1* and *zmHKT1* was detected in plants bioprimed with bioinoculants alone or in combination. These results were positively correlated with increased salt tolerance. In the present study, we found that the expression of *zmNHX1 and zmHKT1* was up-regulated in the roots of maize plants bioprimed with microbial inoculant(s) and exposed to salt concentrations in the soil. In untreated plants, the expression of *zmNHX1* and *zmHKT1* is usually very weak. Thus, the above studies involving microbial inoculation confirmed this point. These results suggested that Na^+^ compartmentalization is a crucial factor and plays an important role in the roots during early establishment of crop plants under salt stress. The transport of Na^+^ into the vacuoles of the roots was likely facilitated by tonoplast Na^+^/H^+^ antiporter encoded by *zmNHX1* genes under high salinity, which prevented the toxic effect of Na^+^ to the plant system (Jiang et al., 2016; Zhang et al., 2017). The present study, showed novel insights into microbe-mediated mechanisms of root architecture development, expression of several genes related to lateral root development, antioxidant enzymes, Na^+^/K^+^ transporter, effects of microbial inoculation on soil physio-biochemical properties, and crop establishment under salt stress in detail. However, the exact mechanisms of microbe-mediated Na^+^ compartmentalization and regulation of physiological and metabolic pathways in maize are still unclear, and the molecular mechanism needs to be further analyzed in the future. Further, it would be interesting to explore how the overexpression of *zmNHX1*, *zmHKT1*, and other antioxidant genes could be used to enhance salt tolerance and better crop growth in maize, similar to achievements made with *A. thaliana* and other plants.

## Conclusion

The present study reveals that seed biopriming and microbial inventerization restructured the cellular responses that provide early establishment, ecological fitness, salt tolerance, and adaptiveness to the maize grown in saline–sodic soil. The increased number of lateral roots and vigorous growth of shoots was discovered to be phenotypic adaptation and the higher accumulation of compatible solutes, phenolics, flavonoids, and reduced ROS contributed to the enhanced salt tolerance in the maize plants bioprimed with either of strains or in combination. A reduced Na^+^ content and increase in K^+^ and Ca^2+^ were positively correlated with an increased transcript level of Na^+^/K^+^ transporter in plants. The highly expressed *ZmHO-1*, *ZmGSL-1*, *ZmGSL-3*, *zmAPx-1.2*, *zmBADH-1*, *zmCAT*, *zmMPK-5*, *zmMPK-7*, *zmCPK-11*, *zmNHX1*, and *zmHKT1* genes could play important roles in lateral root development, antioxidant properties, and ion homeostasis at the cellular level. Moreover, this study provides new insights into the molecular mechanism of microbe-mediated salt tolerance and could lay a foundation for crop management under salt stress. Furthermore, analyses of the soil enzymes and uptake and translocation of Na^+^ revealed that some Na^+^/K^+^ transporters responded positively to microbial inoculation and salt stress, suggesting that these transporters are probably key molecular targets of microbe-mediated salt tolerance in maize. Further, this study clearly indicated that biological interactions between beneficial microorganisms and roots of maize plants could be an alternative for salt stress management in maize.

The present investigation tried to elucidate the microbe-mediated mechanisms of salt tolerance, but a key knowledge gap remains unclear, especially regarding metabolome and metatranscriptome changes during salt stress in maize. The present study is a step ahead from the current knowledge on plant–microbe interactions under salt stress. Going beyond the simple biopriming and interaction study, a comprehensive and multi-omics based study is needed to analyze the physiological responses with proper validation and testing of hypothesis *via in vitro* and *in vivo* experiments and should be the critical next step. Herein, based on observation and recent research, we proposed a multi-disciplinary system-based approach that includes plant genetics, physiology, soil science, and plant biotechnology to better understand the plant–microbe interactions with special reference to salt/abiotic stress tolerance for improving agricultural productivity and environmental sustainability.

## Data Availability Statement

The original contributions presented in the study are included in the article/Supplementary Material, further inquiries can be directed to the corresponding author/s.

## Author Contributions

SS, US, PSh, MT, and AS conceived and designed the experiments. SS, US, DM, MM, and PKS performed the experiments. US, HS, and PKS analyzed the data. MR did the SEM. SS, US, HS, and MM wrote the manuscript. All authors have reviewed the manuscript and have given approval to the final version.

## Conflict of Interest

The authors declare that the research was conducted in the absence of any commercial or financial relationships that could be construed as a potential conflict of interest.
