# Supplementary material for: Restructuring the Cellular Responses: Connecting Microbial Intervention With Ecological Fitness and Adaptiveness to the Maize (Zea mays L.) Grown in Saline–Sodic Soil
Source: Front Microbiol. 2021 Feb 12;11:568325. doi: 10.3389/fmicb.2020.568325 (PMC7907600; doi:10.3389/fmicb.2020.568325)
Supplement: Supplementary file 1 [file Data_Sheet_1.doc]

**Supplementary Table 1.** Geographical location and enumeration of rhizospheric bacteria strains isolated from different parts of Uttar Pradesh, India.

| Strain name | Place of isolation | Coordinates | Plant growth stage | Soil type |
| --- | --- | --- | --- | --- |
| MF-01 | Devapur, Ghazipur, Uttar Pradesh (India) | 25°46’45.63” N 83°32’32.05” E | V-5 stage | Silt loam |
| MF-02 | Govindpur, Ghazipur, Uttar Pradesh (India) | 25°46’34.75” N 83°33’48.99” E | V-5 stage | Silt loam |
| MF-03 | Govindpur Kirat, Ghazipur, Uttar Pradesh (India) | 25°45’58.15” N 83°33’48.77” E | V-5 stage | Silt loam |
| MF-04 | Shakkapur, Ghazipur, Uttar Pradesh (India) | 25°47’22.38” N 83°34’13.57” E | V-9 stage | Silt loam |
| MF-04-1 | Indaur, Ghazipur, Uttar Pradesh (India) | 25°47’08.77” N 83°36’30.51” E | V-9 stage | Silt loam |
| MF-06 | Tejpura, Ghazipur, Uttar Pradesh (India) | 25°47’35.45” N 83°35’59.99” E | V-9 stage | Silt clay loam |
| MF-08 | Bhawanipur, Mardah, Ghazipur, Uttar Pradesh (India) | 25°48’56.19” N 83°33’15.41” E | V-15 stage | Silt clay loam |
| MF-10 | Nasaratpur, Ghazipur, Uttar Pradesh (India) | 25°44’48.69” N 83°32’40.31” E | V-15 stage | Silt clay loam |
| MF-11 | Bhawanipur, Mardah, Ghazipur, Uttar Pradesh (India) | 25°48’56.19” N 83°33’15.41” E | V-15 stage | Silt clay loam |
| MF-12 | Bhawanipur, Mardah, Ghazipur, Uttar Pradesh (India) | 25°48’56.19” N 83°33’15.41” E | V-15 stage | Silt clay loam |
| MF-15 | Devapur, Ghazipur, Uttar Pradesh (India) | 25°46’45.63” N 83°32’32.05” E | V-15 stage | Silt loam |
| MF-21 | Govindpur, Ghazipur, Uttar Pradesh (India) | 25°46’34.75” N 83°33’48.99” E | Tasseling stage | Silt loam |
| MF-25 | Govindpur Kirat, Ghazipur, Uttar Pradesh (India) | 25°45’58.15” N 83°33’48.77” E | Tasseling stage | Silt loam |
| MF-30 | Paniyara, Mau, Uttar Pradesh (India) | 25°53’48.19” N 83°31’52.27” E | Tasseling stage | Silt clay loam |
| MF-35 | Kushmaur, Mau, Uttar Pradesh (India) | 25°54’22.32” N 83°29’36.86” E | Tasseling stage | Silt loam |
| MF-41 | Kanjha Khurd, Mau, Uttar Pradesh (India) | 25°57’26.13” N 83°30’02.68” E | Silking stage | Silt loam |
| MF-49 | Ekauna, Mau, Uttar Pradesh (India) | 26°00’55.93” N 83°29’01.02” E | Silking stage | Silt loam |
| MF-52 | Gauharpur, Mau, Uttar Pradesh (India) | 26°01’35.08” N 83°28’47.38” E | Silking stage | Silt clay loam |
| MF-53 | Bhawanipur, Mardah, Ghazipur, Uttar Pradesh (India) | 25°48’56.19” N 83°33’15.41” E | Silking stage | Silt clay loam |
| MF-54 | Palahipur, Ghazipur, Uttar Pradesh (India) | 25°46’14.79” N 83°31’56.28” E | Silking stage | Silt clay loam |
| MF-57 | Chaubepur, Ghazipur, Uttar Pradesh (India) | 25°46’18.13” N 83°28’49.59” E | Milk stage | Silt clay loam |
| MF-58 | Dullahpur Ghazipur Uttar Pradesh (India) | 25°50’41.89” N 83°25’05.25” E | Milk stage | Clay loam |
| MF-59 | Girija Bhavan Ballia, Uttar Pradesh (India) | 25°49’55.76” N 84°13’34.80” E | Milk stage | Clay loam |
| MF-60 | Bhawanipur, Mardah, Ghazipur, Uttar Pradesh (India) | 25°48’56.19” N 83°33’15.41” E | Dough stage | Silt clay loam |
| MF-61 | KVK, Ghazipur Uttar Pradesh (India) | 25°32’40.13” N 83°24’06.53” E | Dough stage | Silt loam |
| MF-62 | Dullahpur Ghazipur Uttar Pradesh (India) | 25°50’41.89” N 83°25’05.25” E | Dough stage | Clay loam |
| MF-65 | Mardah Ghazipur Uttar Pradesh (India) | 25°48’59.11” N 83°33’00.96” E | Dough stage | Silt clay loam |
| MF-66 | Nasaratpur, Ghazipur, Uttar Pradesh (India) | 25°44’48.69” N 83°32’40.31” E | Dough stage | Silt clay loam |
| MF-67 | Nasaratpur, Ghazipur, Uttar Pradesh (India) | 25°44’48.69” N 83°32’40.31” E | V-15 stage | Silt clay loam |
| MF-68 | KVK, Ghazipur Uttar Pradesh (India) | 25°32’40.13” N 83°24’06.53” E | V-15 stage | Silt loam |
| MF-69 | Kanjha Khurd, Mau, Uttar Pradesh (India) | 25°57’26.13” N 83°30’02.68” E | V-15 stage | Silt loam |
| MF-72 | Ekauna, Mau, Uttar Pradesh (India) | 26°00’55.93” N 83°29’01.02” E | V-15 stage | Silt loam |
| MF-72-1 | Gauharpur, Mau, Uttar Pradesh (India) | 26°01’35.08” N 83°28’47.38” E | Tasseling stage | Silt clay loam |
| MF-74 | Shakkapur, Ghazipur, Uttar Pradesh (India) | 25°47’22.38” N 83°34’13.57” E | Tasseling stage | Silt loam |
| MF-75 | Indaur, Ghazipur, Uttar Pradesh (India) | 25°47’08.77” N 83°36’30.51” E | Tasseling stage | Silt loam |
| MF-75-1 | Tejpura, Ghazipur, Uttar Pradesh (India) | 25°47’35.45” N 83°35’59.99” E | Tasseling stage | Silt clay loam |
| MF-76 | Govindpur Kirat, Ghazipur, Uttar Pradesh (India) | 25°45’58.15” N 83°33’48.77” E | Silking stage | Silt loam |
| MF-78 | Paniyara, Mau, Uttar Pradesh (India) | 25°53’48.19” N 83°31’52.27” E | Silking stage | Silt clay loam |
| MF-79 | KVK, Ghazipur Uttar Pradesh (India) | 25°32’40.13” N 83°24’06.53” E | Silking stage | Silt loam |
| MF-80 | Bhauraha, Ghazipur, Uttar Pradesh (India) | 25°43’47.17” N 83°31’31.73” E | Silking stage | Silt clay loam |
| MF-81 | Baddhoopur, Ghazipur, Uttar Pradesh (India) | 25°44’47.31” N 83°28’55.30” E | V-5 stage | Clay loam |
| MF-83 | Chaubepur, Ghazipur, Uttar Pradesh (India) | 25°46’18.13” N 83°28’49.59” E | V-5 stage | Silt clay loam |
| MF-84 | Bahlolpur, Ghazipur, Uttar Pradesh (India) | 25°49’33.73” N 83°28’44.48” E | V-9 stage | Silt loam |
| MF-85 | Nakhatpur, Ghazipur, Uttar Pradesh (India) | 25°51’02.28” N 83°29’34.32” E | V-9 stage | Silt loam |
| MF-86 | Govindpur, Ghazipur, Uttar Pradesh (India) | 25°46’34.75” N 83°33’48.99” E | V-9 stage | Silt loam |
| MF-86-1 | Govindpur Kirat, Ghazipur, Uttar Pradesh (India) | 25°45’58.15” N 83°33’48.77” E | V-15 stage | Silt loam |
| MF-88 | Paniyara, Mau, Uttar Pradesh (India) | 25°53’48.19” N 83°31’52.27” E | V-15 stage | Silt clay loam |
| MF-89 | KVK, Ghazipur Uttar Pradesh (India) | 25°32’40.13” N 83°24’06.53” E | Silking stage | Silt loam |
| MF-90 | KVK, Ghazipur Uttar Pradesh (India) | 25°32’40.13” N 83°24’06.53” E | Silking stage | Silt loam |
| MF-101 | Chaubepur, Ghazipur, Uttar Pradesh (India) | 25°46’18.13” N 83°28’49.59” E | Dough stage | Silt loam |

**Supplementary Table 2.** The physico-chemical properties of initial experimental soil

| **S. No.** | **Soil properties** | **Value** |
| --- | --- | --- |
| 1. | Soil separates^a^ (%) |  |
| (i) | Coarse sand (0.2-2.0 mm) | 12.22 |
| (ii) | Fine sand (0.02-0.2 mm) | 18.17 |
| (iii) | Silt (0.002-0.02mm) | 47.50 |
| (iv) | Clay(< 0.002 mm) | 22.75 |
| 2. | Textural class | Silt loam |
| 3. | pH^b^ | 8.2 |
| 4. | EC (dS m^-1^) | 2.75 |
| 5. | OC (g kg^-1^) | 5.33 |
| 6. | OM (g kg^-1^) | 8.24 |
| 7. | Bulk density (Mg m^-3^) | 1.50 |
| 8. | Particle density (Mg m^-3^) | 2.35 |
| 9. | Total CEC^c^ [cmol (P^+^) kg^-1^] | 13.66 |
| 10. | CaCO_3_ (%) | 7.50 |
| 11. | Available macronutrients (kg ha^-1^) | |
| (i) | N | 201.66 |
| (ii) | P | 46.50 |
| (iii) | K | 150.33 |
| (iv) | S | 7.75 |
| 12. | Available micronutrients (mg kg^-1^) | |
| (i) | Fe | 10.25 |
| (ii) | Mn | 2.75 |
| (iii) | Cu | 0.85 |
| (iv) | Zn | 0.66 |
| (v) | B | 0.10 |
| (vi) | Mo | 0.10 |

^a^ Hydrometer methods, ^b^ Water:soil= 2.5:1, ^c^ Method: 1 M NH4 acetate extractable cations with prewash, EC - Electrical conductivity, OC - organic carbon, OM - organic matter, CEC- cation exchange capacity

**Supplementary Table 3.** Screening of bacterial strains for their PGP and biochemical traits.

| Strain name | Species name | Biochemical parameters | | | | | | | | | | |
| --- | --- | --- | --- | --- | --- | --- | --- | --- | --- | --- | --- | --- |
|  |  | Sidrophore | HCN | IAA | Ammonia | P | K | Zn | Protease | Amylase | Cellulase | Pectinase |
| MF-01 | *Bacillus safensis* | +++ | + | +++ | +++ | +++ | ++ | ++ | ++ | + | + | + |
| MF-02 | *Entero bacter cloacae* subsp*. dissolvens* | ++ | - | +++ | ++ | + | + |  | - | ++ | - | - |
| MF-03 | *Bacillus safensis* | +++ | - | + | ++ | ++ | ++ | ++ | ++ | + | + | + |
| MF-04 | *Bacillus aryabhattai* | + | - | +++ | ++ | + | + | + | +++ | - | - | - |
| MF-04-1 | *Bacillus megatetium* | + | - | + | + | + | - | + | ++ | - | - | - |
| MF-06 | *Bacillus altitudinis* | +++ | - | +++ | +++ | ++ | ++ | ++ | +++ | ++ | ++ | ++ |
| MF-08 | *Bacillus velezensis* | +++ | + | +++ | +++ | +++ | +++ | +++ | +++ | ++ | ++ | +++ |
| MF-10 | *Bacillus bingmayongensis* | + | - | ++ | + | + | - | - | ++ | - | - | - |
| MF-11 | *Bacillus aryabhattai* | + | - | + | +++ | + | - | + | +++ | - | - | - |
| MF-12 | *Bacillus aryabhattai* | + | - | ++ | ++ | + | - | + | +++ | - | - | - |
| MF-15 | *Bacillus altitudinis* | +++ | + | +++ | ++ | +++ | +++ | +++ | +++ | + | + | ++ |
| MF-21 | *Bacillus manliponesis* | ++ | - | + | + | + | - | - | ++ | - | +++ | - |
| MF-25 | *Bacillus tequilensis* | + | + | +++ | ++ | ++ | - | - | +++ | - | - | - |
| MF-30 | *Pseudomonas aeruginosa* | +++ | +++ | +++ | +++ | +++ | ++ | +++ | +++ | ++ | - | ++ |
| MF-35 | *Bacillus oceanisediminis* | + | - | ++ | ++ | + | - | + | ++ | + | + | - |
| MF-41 | *Bacillus paramycoides* | + | - | + | ++ | + | + | + | ++ | + | - | - |
| MF-49 | *Bacillus flexus* | + | - | + | + | - | - | - | + | - | - | - |
| MF-52 | *Bacillus bingmayongensis* | + | + | +++ | + | + | - | - | + | + | - | + |
| MF-53 | *Bacillus altitudinis* | +++ | - | + | ++ | +++ | +++ | +++ | +++ | + | ++ | + |
| MF-54 | *Bacillus safensis* | +++ | - | + | +++ | ++ | ++ | +++ | +++ | ++ | + | - |
| MF-57 | *Bacillus cereus* | ++ | - | + | + | - | - | - | + | - | ++ | + |
| MF-58 | *Bacillus altitudinis* | +++ | - | +++ | ++ | +++ | +++ | +++ | ++ | + | ++ | +++ |
| MF-59 | *Bacillus altitudinis* | +++ | - | +++ | ++ | +++ | +++ | +++ | ++ | + | ++ | ++ |
| MF-60 | *Bacillus paralicheniformis* | + | - | + | + | - | - | - | + | - | - | - |
| MF-61 | *Bacillus cereus* | ++ | - | + | + | - | - | - | - | + | - | - |
| MF-62 | *Bacillus simplex* | + | - | ++ | + | - | - | + | + | - | + | - |
| MF-65 | *Bacillus altitudinis* | +++ | - | +++ | +++ | +++ | +++ | +++ | ++ | +++ | - | - |
| MF-66 | *Bacillus aryabhattai* | ++ | - | ++ | + | + | + | + | + | - | + | - |
| MF-67 | *Bacillus altitudinis* | +++ | - | +++ | ++ | +++ | +++ | ++ | ++ | ++ | + | ++ |
| MF-68 | *Bacillus pseudomycoides* | + | - | + | + | - | - | + | - | - | + | - |
| MF-69 | *Bacillus cereus* | ++ | - | + | ++ | - | - | - | - | - | - | - |
| MF-72 | *Bacillus bingmayongensis* | + | - | + | - | - | + | - | + | - | - | - |
| MF-72-1 | *Lysinibacillus macroides* | + | - | ++ | + | - | - | - | ++ | - | + | - |
| MF-74 | *Bacillus safensis* | +++ | - | ++ | ++ | +++ | ++ |  | +++ | ++ | ++ | ++ |
| MF-75 | *Bacillus subtilis* subsp*. spizizenii* | ++ | - | + | - | - | - | - |  |  |  |  |
| MF-75-1 | *Bacillus aryabhattai* | ++ | - | ++ | + | + | + | - | + | - | - | - |
| MF-76 | *Bacillus altitudinis* | +++ | - | +++ | +++ | +++ | ++ | ++ | +++ | ++ | ++ | + |
| MF-78 | *Bacillus subtilis* subsp*. stercoris* | ++ | - | + | + | - | - | - | + | - | - | + |
| MF-79 | *Bacillus safensis* | +++ | - | +++ | ++ | ++ | +++ | +++ | +++ | - | + | - |
| MF-80 | *Bacillus firmus* | + | - | + | + | - | - | + | + | - | - | - |
| MF-81 | *Bacillus tequilensis* | + | - | ++ | ++ | + | - | - | ++ | - | - | - |
| MF-83 | *Bacillus tequilensis* | + | - | ++ | ++ | + | - | - | ++ | - | - | - |
| MF-84 | *Pseudomonas geniculata* | +++ | +++ | +++ | ++ | + | ++ | +++ | +++ | +++ | ++ | + |
| MF-85 | *Bacillus halotolerans* | ++ | - | + | + | - | - | - | ++ | - | + | + |
| MF-86 | *Bacillus subtilis* subsp*. spizizenii* | ++ | - | + | - | - | - | - | + | - | - | - |
| MF-86-1 | *Bacillus safensis* | +++ | - | ++ | + | +++ | +++ | +++ | +++ | ++ | +++ | - |
| MF-88 | *Bacillus safensis* | +++ | - | ++ | + | +++ | +++ | ++ | +++ | - | ++ | - |
| MF-89 | *Bacillus subtilis* subsp*. stercoris* | ++ | - | + | - | - | - | - | + | - | - | - |
| MF-90 | *Bacillus aryabhattai* | + | - | + | + | + | + | - | ++ | - | - | - |
| MF-101 | *Bacillus altitudinis* | +++ | - | +++ | ++ | +++ | +++ | +++ | +++ | + | ++ | + |

**Supplementary Table 4.** Identification of bacterial stains with their matching type strain and accession numbers received from NCBI GenBank.

| Strain name | Species name | Accession no. | Matching Type strain |
| --- | --- | --- | --- |
| MF-01 | *Bacillus safensis* | MH 177230 | *Bacillus safensis* FO-36b (T) ASJD01000027 |
| MF-02 | *Enterobacter cloacae* subsp*. dissolvens* | MH 177231 | *Enterobacter cloacae* subsp. *dissolvens* LMG2683 (T) Z96079 |
| MF-03 | *Bacillus safensis* | MH 177232 | *Bacillus safensis* FO-36b (T) ASJD01000027 |
| MF-04 | *Bacillus aryabhattai* | MH 177234 | *Bacillus aryabhattai* B8W22 (T) EF114313 |
| MF-04-1 | *Bacillus megatetium* | MH 177233 | *Bacillus megatetium* NBRC15308 (T) JJMH01000057 |
| MF-06 | *Bacillus altitudinis* | MH 177235 | *Bacillus altitudinis* 41KF2b (T) ASJC01000029 |
| MF-08 | *Bacillus velezensis* | MH 177236 | *Bacillus velezensis* CR-502 (T) AY603658 |
| MF-10 | *Bacillus bingmayongensis* | MH 177237 | *Bacillus bingmayongensis* FJAT-13831 (T) AKCS01000011 |
| MF-11 | *Bacillus aryabhattai* | MH 177238 | *Bacillus aryabhattai* B8W22 (T) EF114313 |
| MF-12 | *Bacillus aryabhattai* | MH 177239 | *Bacillus aryabhattai* B8W22 (T) EF114313 |
| MF-15 | *Bacillus altitudinis* | MH 177240 | *Bacillus altitudinis* 41KF2b (T) ASJC01000029 |
| MF-21 | *Bacillus manliponesis* | MH 177241 | *Bacillus manliponesis* BL4-6 (T) FJ416490 |
| MF-25 | *Bacillus tequilensis* | MH 177242 | *Bacillus subtilis* subsp. *inaquosorum* KCTC13429 (T) AMXN01000021 |
| MF-30 | *Pseudomonas aeruginosa* | MH 177243 | *Pseudomonas aeruginosa* JCM5962 (T) BAMA01000316 |
| MF-35 | *Bacillus oceanisediminis* | MH 177244 | *Bacillus oceanisediminis* H2 (T) GQ292772 |
| MF-41 | *Bacillus paramycoides* | MH 177245 | *Bacillus paranthracis* Mn5 (T) MACE01000012 |
| MF-49 | *Bacillus flexus* | MH 177246 | *Bacillus flexus* NBRC15715 (T) BCVD01000224 |
| MF-52 | *Bacillus bingmayongensis* | MK120869 | *Bacillus bingmayongensis* FJAT-13831 (T) AKCS01000011 |
| MF-53 | *Bacillus altitudinis* | MK120870 | *Bacillus altitudinis* 41KF2b (T) ASJC01000029 |
| MF-54 | *Bacillus safensis* | MH177247 | *Bacillus safensis* FO-36b (T) ASJD01000027 |
| MF-57 | *Bacillus cereus* | MK120871 | *Bacillus cereus* ATCC14579 (T) AE016877 |
| MF-58 | *Bacillus altitudinis* | MK120872 | *Bacillus altitudinis* 41KF2b (T) ASJC01000029 |
| MF-59 | *Bacillus altitudinis* | MK120873 | *Bacillus altitudinis* 41KF2b (T) ASJC01000029 |
| MF-60 | *Bacillus paralicheniformis* | MK120874 | *Bacillus paralicheniformis* KJ-16 (T) KY694465 |
| MF-61 | *Bacillus cereus* | MK120875 | *Bacillus cereus* ATCC14579 (T) AE016877 |
| MF-62 | *Bacillus simplex* | MK120876 | *Bacillus simplex* NBRC15720 (T) BCVO01000086 |
| MF-65 | *Bacillus altitudinis* | MK120877 | *Bacillus altitudinis* 41KF2b (T) ASJC01000029 |
| MF-66 | *Bacillus aryabhattai* | MK120878 | *Bacillus aryabhattai* B8W22 (T) EF114313 |
| MF-67 | *Bacillus altitudinis* | MH 177249 | *Bacillus altitudinis* 41KF2b (T) ASJC01000029 |
| MF-68 | *Bacillus pseudomycoides* | MK120879 | *Bacillus pseudomycoides* DSM12442 (T) ACMX01000133 |
| MF-69 | *Bacillus cereus* | MK120880 | *Bacillus cereus* ATCC14579 (T) AE016877 |
| MF-72 | *Bacillus bingmayongensis* | MH 177250 | *Bacillus bingmayongensis* FJAT-13831 (T) AKCS01000011 |
| MF-72-1 | *Lysinibacillus macroides* | MK120881 | *Lysinibacillus macroides* DSM54 (T) LGCI01000008 |
| MF-74 | *Bacillus safensis* | MK120882 | *Bacillus safensis* FO-36b (T) ASJD01000027 |
| MF-75 | *Bacillus subtilis* subsp*.spizizenii* | MH 177251 | *Bacillus* *subtilis* subsp. *spizizenii* strain EB7, MK184212.1 |
| MF-75-1 | *Bacillus aryabhattai* | MK120883 | *Bacillus aryabhattai* B8W22 (T) EF114313 |
| MF-76 | *Bacillus altitudinis* | MK120884 | *Bacillus altitudinis* 41KF2b (T) ASJC01000029 |
| MF-78 | *Bacillus subtilis* subsp*. stercoris* | MK120885 | *Bacillus subtilis* subsp. *stercoris* D7XPN1(T) JHCA01000027 |
| MF-79 | *Bacillus safensis* | MK120886 | *Bacillus safensis* FO-36b (T) ASJD01000027 |
| MF-80 | *Bacillus firmus* | MK120887 | *Bacillus firmus*,NBRC 15306 (T) BCUY01000205 |
| MF-81 | *Bacillus tequilensis* | MK120888 | *Bacillus subtilis* subsp. *inaquosorum* KCTC13429 (T) AMXN01000021 |
| MF-83 | *Bacillus tequilensis* | MK120889 | *Bacillus subtilis* subsp. *inaquosorum* KCTC13429 (T) AMXN01000021 |
| MF-84 | *Pseudomonas geniculata* | MK120890 | *Pseudomonas geniculata* ATCC19374 (T) AB021404 |
| MF-85 | *Bacillus halotolerans* | MK120891 | *Bacillus halotolerans* ATCC25096 (T) LPVF01000003 |
| MF-86 | *Bacillus subtilis* subsp*. spizizenii* | MH 177252 | *Bacillus* *subtilis* subsp. *spizizenii* strain EB7, MK184212.1 |
| MF-86-1 | *Bacillus safensis* | MK120892 | *Bacillus safensis* FO-36b (T) ASJD01000027 |
| MF-88 | *Bacillus safensis* | MH 177253 | *Bacillus safensis* FO-36b (T) ASJD01000027 |
| MF-89 | *Bacillus subtilis* subsp*. stercoris* | MK120893 | *Bacillus subtilis* subsp. *stercoris* D7XPN1 (T) JHCA01000027 |
| MF-90 | *Bacillus aryabhattai* | MH 177254 | *Bacillus aryabhattai* B8W22 (T) EF114313 |
| MF-101 | *Bacillus altitudinis* | MH 177255 | *Bacillus altitudinis* 41KF2b (T) ASJC01000029 |
